# Nitrogen fixation catalyzed by ferrocene-substituted dinitrogen-bridged dimolybdenum–dinitrogen complexes: unique behavior of ferrocene moiety as redox active site†
†Electronic supplementary information (ESI) available. CCDC 1048952–1048956. For ESI and crystallographic data in CIF or other electronic format see DOI: 10.1039/c5sc00545k


**DOI:** 10.1039/c5sc00545k

**Published:** 2015-04-20

**Authors:** Shogo Kuriyama, Kazuya Arashiba, Kazunari Nakajima, Hiromasa Tanaka, Kazunari Yoshizawa, Yoshiaki Nishibayashi

**Affiliations:** a Institute of Engineering Innovation , School of Engineering , The University of Tokyo , Yayoi, Bunkyo-ku , Tokyo 113-8656 , Japan . Email: ynishiba@sogo.t.u-tokyo.ac.jp; b Elements Strategy Initiative for Catalysts and Batteries (ESICB) , Kyoto University , Nishikyo-ku , Kyoto 615-8520 , Japan; c Institute for Materials Chemistry and Engineering and International Research Center for Molecular System , Kyushu University , Nishi-ku , Fukuoka 819-0395 , Japan . Email: kazunari@ms.ifoc.kyoshu-u.ac.jp

## Abstract

A series of dinitrogen-bridged dimolybdenum–dinitrogen complexes bearing metallocene-substituted PNP-pincer ligands is synthesized by the reduction of the corresponding monomeric molybdenum–trichloride complexes under 1 atm of molecular dinitrogen. Introduction of ferrocene as a redox-active moiety to the pyridine ring of the PNP-pincer ligand increases the catalytic activity for the formation of ammonia from molecular dinitrogen, up to 45 equiv. of ammonia being formed based on the catalyst (22 equiv. of ammonia based on each molybdenum atom of the catalyst). The time profile for the catalytic reaction reveals that the presence of the ferrocene unit in the catalyst increases the rate of ammonia formation. Electrochemical measurement and theoretical studies indicate that an interaction between the Fe atom of the ferrocene moiety and the Mo atom in the catalyst may play an important role to achieve a high catalytic activity.

## Abstract 2

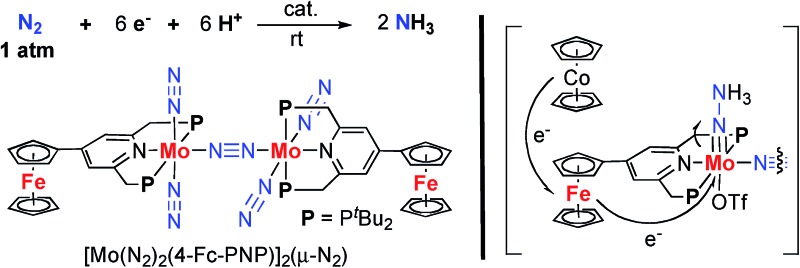
Mo–N_2_ complex bearing ferrocenes as redox-active units efficiently catalyses the formation of ammonia from molecular dinitrogen under ambient conditions.

## 1. Introduction

**Scheme 1:**
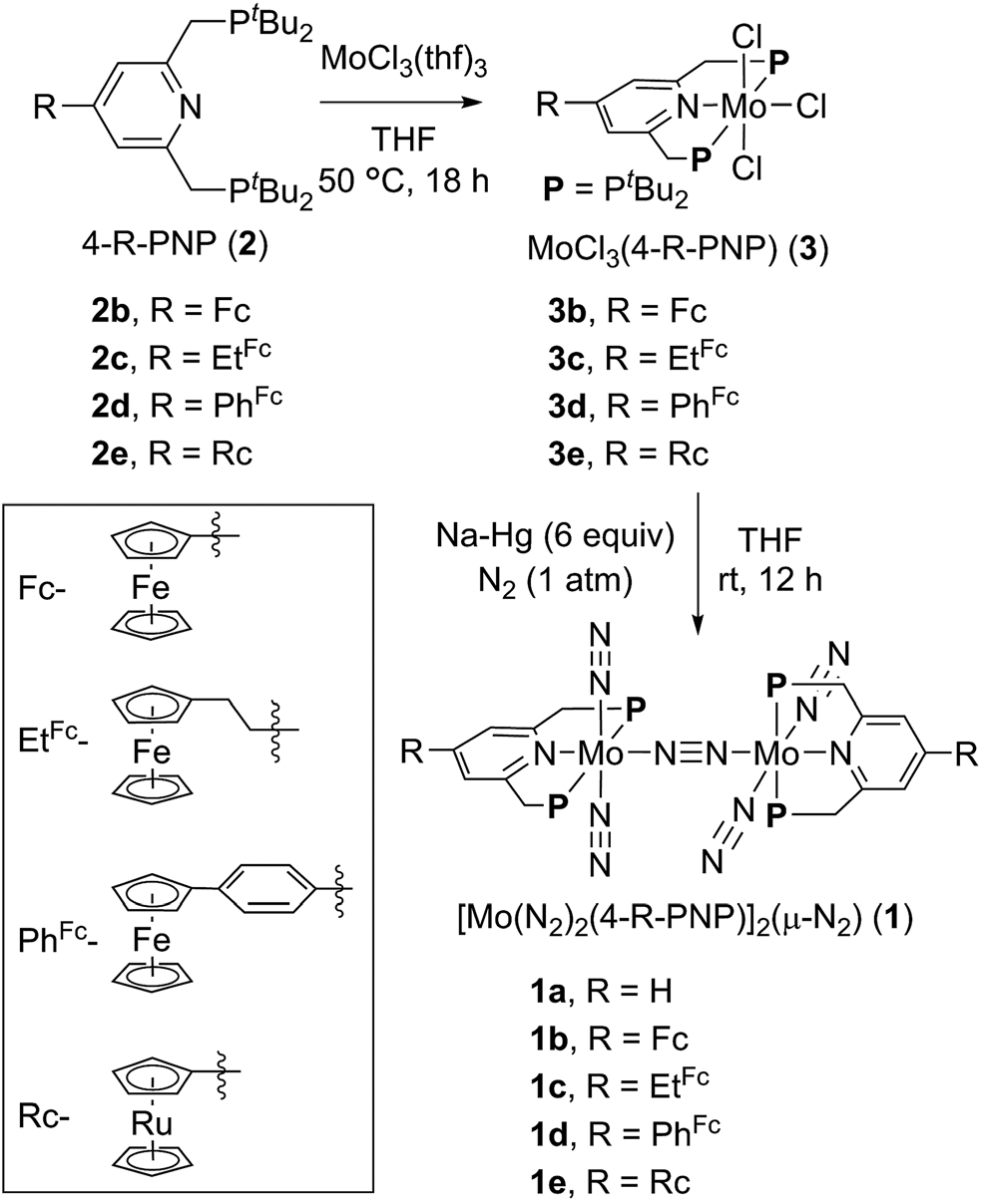
Synthesis of dinitrogen-bridged dimolybdenum–dinitrogen complexes bearing metallocene-substituted PNP-pincer ligands **1**.

Activation and transformation of molecular dinitrogen using transition metal–dinitrogen complexes have been extensively studied to achieve novel nitrogen fixation systems under mild reaction conditions.^1^,^2^ Although a variety of stoichiometric reactivities of transition metal–dinitrogen complexes have been reported upto now, the catalytic conversion of molecular dinitrogen under ambient reaction conditions using these complexes as catalysts has so far been limited to only a few cases.^3^ The first successful example of the catalytic transformation of molecular dinitrogen into ammonia under ambient reaction conditions in the presence of a transition metal–dinitrogen complex was reported by Schrock and co-workers in 2003, where a molybdenum–dinitrogen complex bearing a triamidoamine ligand worked as a catalyst to produce 8 equiv. of ammonia based on the catalyst.^4^ In 2010, we reported another successful example of ammonia formation from molecular dinitrogen by using a dinitrogen-bridged dimolybdenum–dinitrogen complex bearing PNP-pincer ligands [Mo(N_2_)_2_(**PNP**)]_2_(μ-N_2_) (**1a**; **PNP** = 2,6-bis(di-*tert*-butylphosphinomethyl)pyridine) (Scheme 1) as a catalyst.^5^ More recently, Peters and co-workers have found the third successful example by using well-designed iron–dinitrogen complexes as catalysts.^6^

In our reaction system, a theoretical study revealed that a synergistic effect between two Mo moieties of the dinitrogen-bridged dimolybdenum–dinitrogen complex plays an important role to achieve the catalytic activity.^5^,^7^,^8^ An electron transfer from a Mo core to the active site of the other core *via* the bridging dinitrogen ligand contributes to the protonation steps of the coordinated nitrogenous ligands on the Mo atom. On the basis of the proposed catalytic pathway, we have succeeded in the development of a more effective catalyst by tuning the electronic property of the PNP-pincer ligand, where up to 52 equiv. of ammonia based on the catalyst (26 equiv. based on the Mo atom of the catalyst) were produced by using catalysts bearing an electron-donating group at the 4-position of the pyridine ring of the PNP-pincer ligand.^9^ Mechanistic studies suggested that the introduction of an electron-donating group to the PNP-pincer ligand enables a facile protonation on the terminal dinitrogen ligand on the Mo atom and also the suppression of the formation of a side product, molecular dihydrogen.^9^ However, not expectedly, the introduction of an electron-donating group such as methoxy group to the PNP-pincer ligand decreased the rate of ammonia formation probably due to the difficulty in reduction steps involved in the catalytic reaction. These results prompted us to develop a more efficient reaction system, where the reduction steps in the catalytic reaction can also be accelerated by suitably modifying the catalyst.

**Fig. 1:**
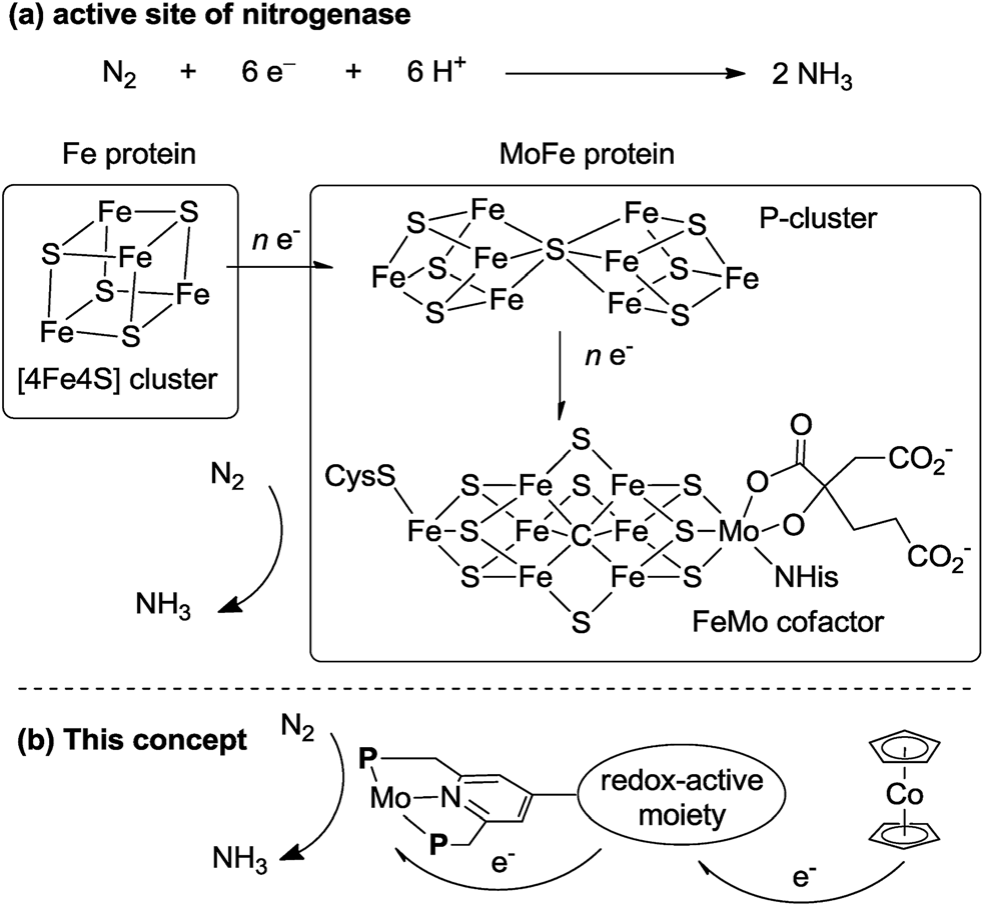
(a) Structure of the 4Fe4S cluster, P-cluster and FeMo cofactor of nitrogenase and an electron flow toward FeMo cofactor. (b) Our concept for a new reaction system by using Mo complexes bearing a redox-active moiety.

**Fig. 2:**
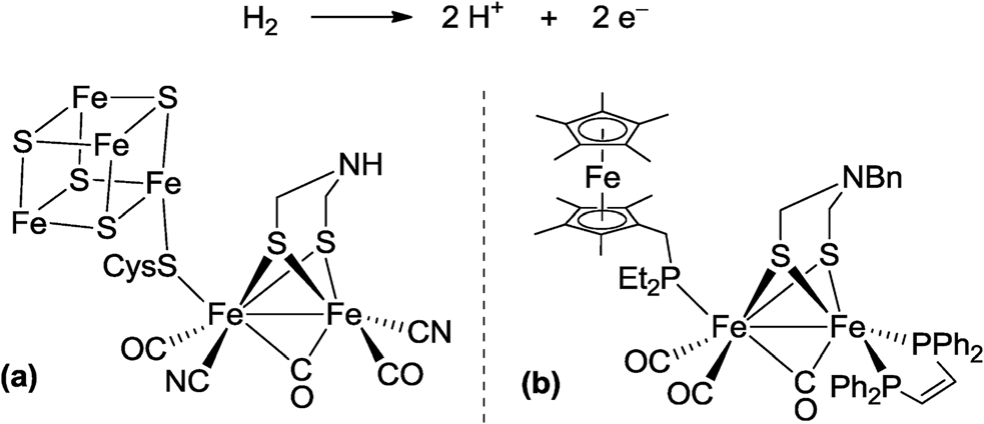
Reaction system of hydrogenase for oxidation of molecular dihydrogen. (a) Active site of [FeFe]hydrogenase. (b) Thiolate-bridged diiron complex bearing a ferrocenylmethylphosphine as a redox-active ligand.

In biological nitrogen fixation, nitrogenase enzymes which are composed of two proteins named the MoFe protein and Fe protein have been known to catalyze ammonia formation from molecular dinitrogen under ambient reaction conditions (Fig. 1).^10^ The active site of the nitrogenase,^11^ known as FeMo cofactor, receives electrons successively from P- and 4Fe4S-clusters, which are located adjacent to FeMo cofactor, to facilitate the multi-electron reduction of molecular dinitrogen into ammonia. By considering the so-far proposed reaction system of nitrogenase, we have envisaged the introduction of a ferrocene unit as a redox-active moiety to the PNP-pincer ligand of the dinitrogen-bridged dimolybdenum–dinitrogen complex, hoping to accelerate the reduction steps involved in the catalytic reaction *via* an intramolecular electron transfer from the Fe atom of ferrocene to the active site of the Mo atom in the complex.^12^ In fact, several research groups have already reported enzyme model complexes bearing a ferrocene unit as a redox-active moiety^13^ because ferrocene is known to be one of the most popular redox-active compounds due to its reversible one-electron redox behavior.^14^,^15^ As a typical example, Camara and Rauchfuss reported that a thiolate-bridged diiron complex bearing a ferrocenylphosphine as a redox-active ligand acted as a [FeFe]hydrogenase model complex (Fig. 2), where an intramolecular electron transfer from the Fe atom in the thiolated-bridged diiron core to the Fe atom of the ferrocene unit plays a critical role to promote the catalytic oxidation of molecular dihydrogen under ambient reaction conditions.13*a*

In this article, we describe the preparation and catalytic activity of a series of dinitrogen-bridged dimolybdenum–dinitrogen complexes bearing a metallocene unit such as ferrocene and ruthenocene (**1**) (Scheme 1). The dinitrogen-bridged dimolybdenum–dinitrogen complexes bearing ferrocene-substituted PNP-pincer ligands have been found to work as efficient catalysts, where up to 45 equiv. of ammonia are produced based on the catalyst (22 equiv. of ammonia based on each Mo atom of the catalyst). The unique behavior of the newly introduced ferrocene unit as a redox-active moiety in the complexes was discussed by means of experimental and theoretical methods.

## 2. Results and discussion

### 2.1. Synthesis of molybdenum complexes bearing metallocene-substituted PNP-pincer ligands

A series of metallocene-substituted PNP-pincer ligands 4-R-PNP (**2**; 4-R-PNP = 4-substituted 2,6-bis(di-*tert*-butylphosphinomethyl)pyridine) was newly prepared (Scheme 1) and the detailed experimental procedure is described in the ESI.† A variety of substituents such as ferrocenyl (Fc), 2-ferrocenylethyl (Et^Fc^), 4-ferrocenylphenyl (Ph^Fc^), and ruthenocenyl (Rc) groups were introduced to the 4-position of pyridine ring of the PNP-pincer ligand. Treatment of [MoCl_3_(thf)_3_] (thf = tetrahydrofuran) with 1 equiv. of 4-R-PNP **2** in tetrahydrofuran (THF) at 50 °C for 18 h gave the corresponding molybdenum–trichloride complexes bearing the corresponding PNP-pincer ligands [MoCl_3_(4-R-PNP)] (**3**) in high yields. The molecular structures of **3** were confirmed by X-ray crystallography (see the ESI in Fig. S3–S5†).

**Fig. 3:**
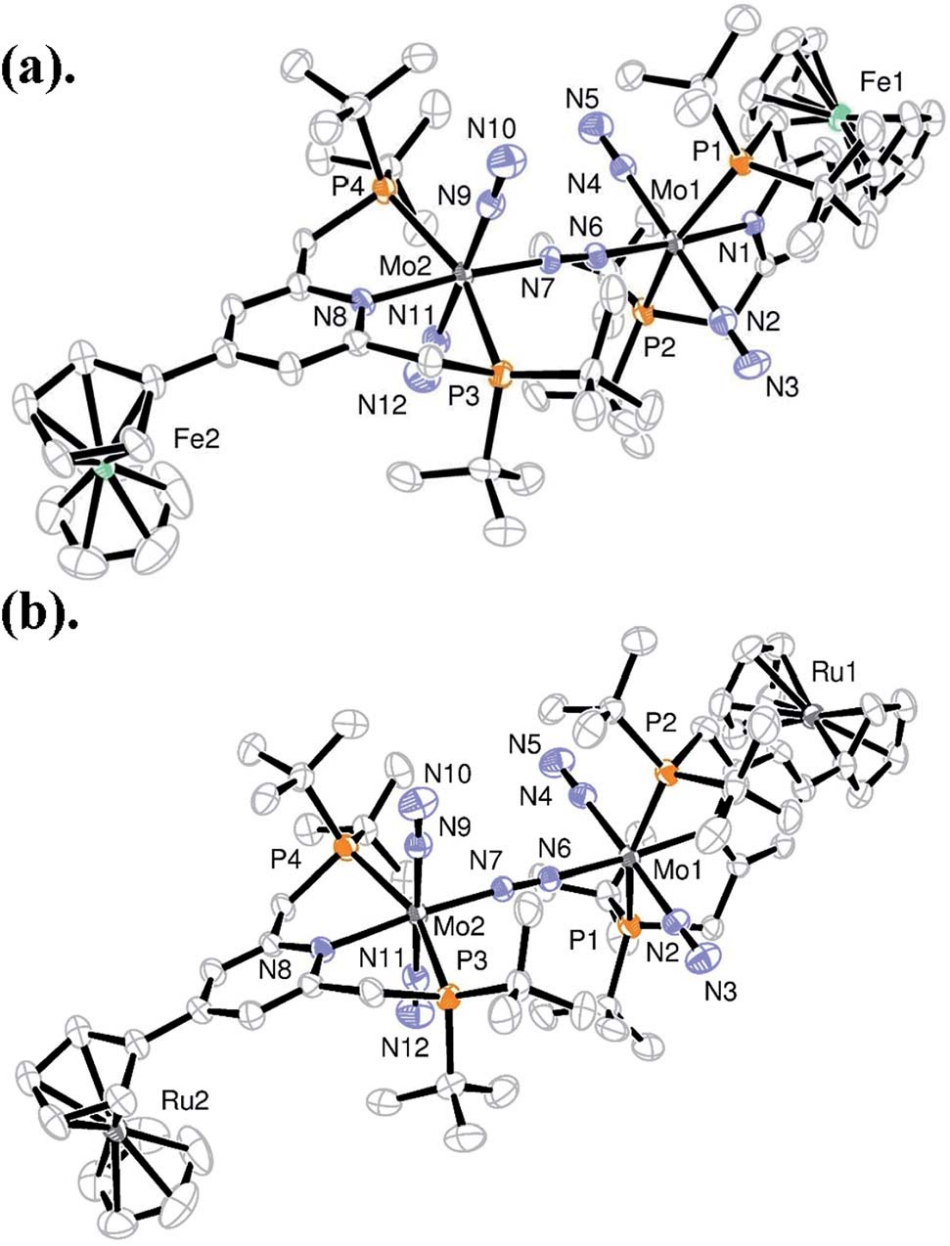
ORTEP drawings of **1b** (a) and **1e** (b). Hydrogen atoms and solvent molecules are omitted for clarity.

Reduction of **3** with 6 equiv. of Na–Hg in THF at room temperature for 12 h under an atmospheric pressure of molecular dinitrogen gave the corresponding metallocene-substituted dinitrogen-bridged dimolybdenum–dinitrogen complexes [Mo(N_2_)_2_(4-R-PNP)]_2_(μ-N_2_) (**1**) in good yields. These new dinitrogen-bridged dimolybdenum–dinitrogen complexes were characterized spectroscopically. Detailed molecular structures of [Mo(N_2_)_2_(4-Fc-PNP)]_2_(μ-N_2_) (**1b**) and [Mo(N_2_)_2_(4-Rc-PNP)]_2_(μ-N_2_) (**1e**) were determined by X-ray crystallography. ORTEP drawings of **1b** and **1e** are shown in Fig. 3. No distinct differences of bond distances and angles were observed in these complexes (Table S9†).

The nature of metallocene as substituent was examined by IR spectroscopy because the electronic nature of substituent at the 4-position in pyridine ring of the PNP pincer-ligand is known to affect absorptions derived from the corresponding terminal dinitrogen ligand.^9^ Typical results are shown in Table 1. The presence of Fc and Rc groups at the 4-position in pyridine ring of the PNP-pincer ligands in **1** did not change the N≡N stretching frequencies (1944 cm^–1^ (**1b**) and 1944 cm^–1^ (**1e**), respectively). These results indicate that the introduction of these metallocene moieties to the pyridine ring of the PNP-pincer ligand would not affect the electronic density on the molybdenum atom in the complex **1**. On the other hand, the N≡N stretching frequency of [Mo(N_2_)_2_(4-Et^Fc^-PNP)]_2_(μ-N_2_) (**1c**) (1939 cm^–1^) is smaller than that of **1a** and the same as that of [Mo(N_2_)_2_(4-Me-PNP)]_2_(μ-N_2_) (**1f**, 1939 cm^–1^).^9^ This result indicates that the Et^Fc^ group has an electron-donating ability as strong as the methyl group in the complex. In contrast, the N≡N frequency of [Mo(N_2_)_2_(4-Ph^Fc^-PNP)]_2_(μ-N_2_) (**1d**) is larger than that of **1a**, and the same as that of [Mo(N_2_)_2_(4-Ph-PNP)]_2_(μ-N_2_) (**1g**, 1950 cm^–1^), indicating that the Ph^Fc^ group acts as an electron-withdrawing substituent in the complex.

**Table 1:** Yield of **1** and IR absorbance of terminal dinitrogen ligand in **1**^*a*^

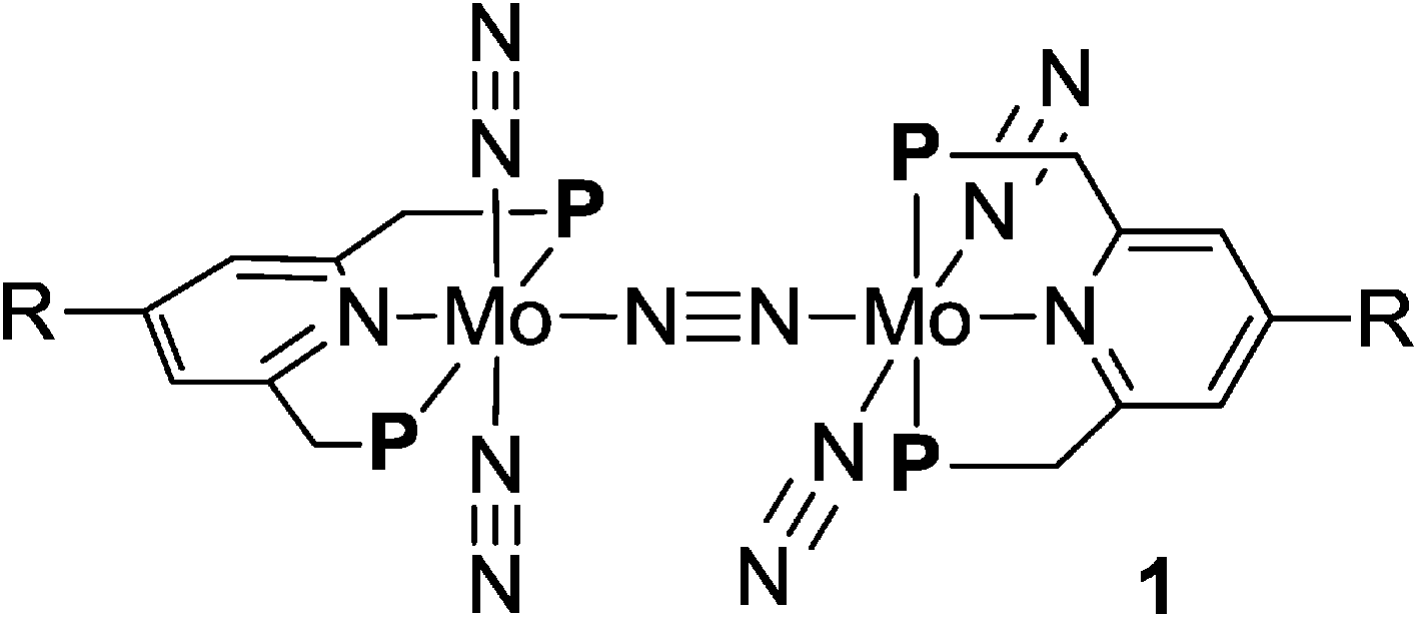			
Complex	R	Yield of **1**^*b*^ (%)	*ν*_NN_/cm^–1^
**1a**	H	63	1944
**1b**	Fc	74	1944
**1c**	Et^Fc^	62	1939
**1d**	Ph^Fc^	32	1951
**1e**	Rc	52	1944
**1f** ^*c*^	Me	—	1939^*c*^
**1g** ^*c*^	Ph	—	1950^*c*^

^*a*^IR absorbance was measured in a THF solution of **1**.

^*b*^Based on **3**.

^*c*^ Ref. 9.

### 2.2. Electrochemical property of molybdenum complexes bearing metallocene-substituted PNP-pincer ligands

**Fig. 4:**
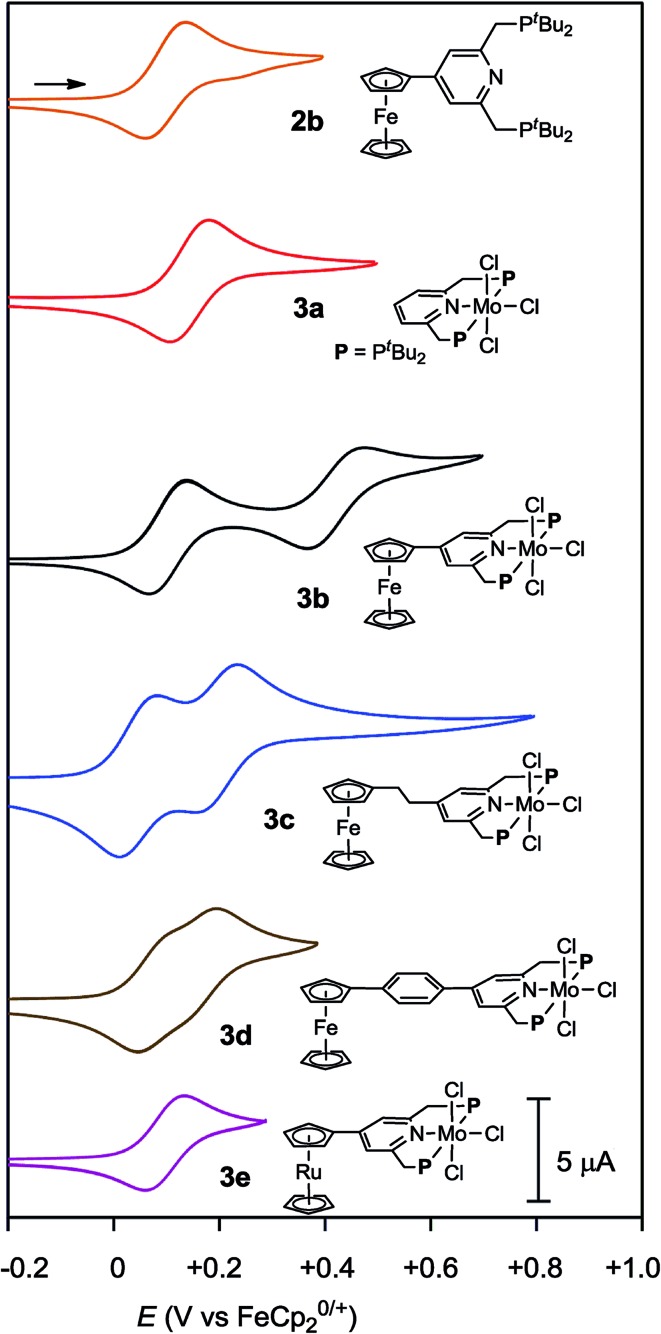
Cyclic voltammograms of **2b** and **3a–e**.

Cyclic voltammograms (CVs) of PNP-pincer ligands **2b–e** in THF with [NBu_4_]BAr^F^_4_ (Ar^F^ = 3,5-(CF_3_)_2_C_6_H_3_) as supporting electrolyte showed oxidation processes assigned to metallocene moieties. Typical results are shown in Table 2. The PNP-pincer ligand bearing a ferrocene moiety, **2b**, showed a reversible one-electron oxidation wave derived from the Fe(ii/iii) couple at *E*_1/2_ = +0.10 V *vs.* FeCp_2_^0/+^ (Fig. 4). The PNP-pincer ligands bearing ferrocene units such as Et^Fc^ and Ph^Fc^ groups, **2c** and **2d**, were oxidized at *E*_1/2_ = –0.03 V (**2c**) and +0.04 V (**2d**), respectively. On the other hand, the PNP-pincer ligand bearing a Rc moiety, **2e**, showed one irreversible oxidation wave at *E*_a_ = +0.79 V.^16^

**Table 2:** Electrochemical data of PNP-pincer ligands **2** and molybdenum-trichloride complexes **3** bearing PNP-pincer ligand^*a*^

Compd.	R	Mo(iii/iv)/V	Fe(ii/iii)/V	Δ*E*(Fe)^*b*^/V
**2b**	Fc	—	+0.10	—
**2c**	Et^Fc^	—	–0.03	—
**2d**	Ph^Fc^	—	+0.04	—
**2e**	Rc	—	+0.79^*c*^	—
**3a**	H	+0.14	—	—
**3b**	Fc	+0.10	+0.42	+0.32
**3c**	Et^Fc^	+0.05	+0.19	+0.22
**3d**	Ph^Fc^	+0.06^*d*^	+0.13^*d*^	+0.09
**3e**	Rc	+0.09^*e*^	—^*e*^	—

^*a*^Measured by cyclic voltammetry in THF at 0.1 V s^–1^ with [NBu_4_]BAr^F^_4_ as supporting electrolyte. Potentials are indicated as *E*_1/2_ values *vs.* FeCp_2_^0/+^.

^*b*^Δ*E*(Fe) is the difference of the Fe(ii/iii) potential between **2** and **3**.

^*c*^ *E*_pa_ value of ruthenocene oxidation.

^*d*^Measured by differential pulse voltammetry (DPV).

^*e*^Oxidation of ruthenocene was not observed in the electrochemical window.

Electrochemical studies were also carried out on the molybdenum–trichloride complexes bearing a substituted PNP-pincer ligand **3**. Typical results are shown in Table 2 and the CVs of **3** are shown in Fig. 4. Non-substituted complex [MoCl_3_(**PNP**)] (**3a**) has one reversible wave at *E*_1/2_ = +0.14 V assignable to Mo(iii/iv), while the CV of molybdenum–trichloride complex bearing a ferrocene-substituted PNP-pincer ligand, **3b**, showed two successive reversible one-electron waves at +0.10 and +0.42 V. We have performed density functional theory (DFT) calculations to assign the observed oxidation waves. The electronic ground state of the cation of **3b** (**[3b]^+^**) is a triplet state, where the Mulliken spin density is highly localized at the Mo atom (1.85). The removal of an electron from **3b** changes the formal charge of the Mo atom from +3 (d^3^) to +4 (d^2^), and thus the oxidation at *E*_1/2_ = +0.10 V is assignable to Mo(iii/iv). On the other hand, the dication of **3b** (**[3b]^2+^**) adopts a quartet state as the ground spin state, where the spin densities at the Mo and Fe atoms are calculated to be 1.82 and 1.27, respectively. This result suggests that the second oxidation should occur at the Fe atom and the oxidation at *E*_1/2_ = +0.42 V is assignable to Fe(ii/iii). In the molybdenum–trichloride complex **3b**, the *E*_1/2_ value of Fe(ii/iii) of the ferrocene moiety shifts by +0.32 V upon coordination. This is evidence of the electronic interaction between the Mo and Fe centers in the complex **3b**. In fact, similar shifts of the *E*_1/2_ values of Fe(ii/iii) of the ferrocene moiety were observed in several transition metal complexes bearing 4-ferrocenylpyridine and 4′-ferrocenyl-2,2′:6′,2′′-terpyridine as ligands, indicating an electronic interaction between the metal and Fe centers in these complexes.^17^,^18^

**Fig. 5:**
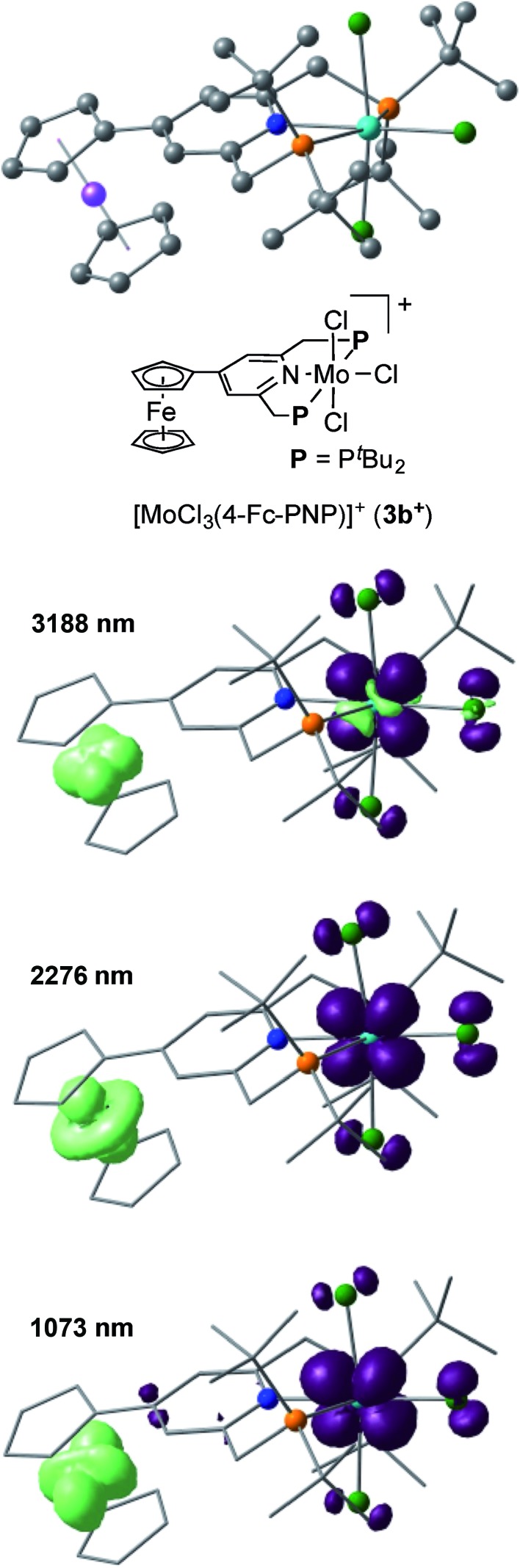
Electron density difference maps (EDDMs) of the electron transitions at 3188, 2276, 1073 nm of **[3b]^+^** calculated with a time-dependent BP86 method. Isosurface plots of loss and gain of electron density are presented in light green and purple, respectively.

The electronic interaction between the Mo and Fe centers in **3b** is also supported by the results of spectroelectrochemical measurements. A weak absorption appeared in the near-IR region at around 800–1800 nm when **3b** was oxidized to **[3b]^+^** (Fig. S8 and S9†). A time-dependent BP86 calculation of **[3b]^+^** predicted three electron transitions at 3188, 2276 and 1073 nm. Fig. 5 depicts electron density difference maps (EDDMs) for these transitions, where EDDMs present isosurface plots of loss (light green) and gain (purple) of electron density for the corresponding electron transitions. Although the observed absorption of **[3b]^+^** in the near IR region was weak, the calculated EDDMs clearly show that these low-energy electron transitions were assignable to the metal-to-metal charge transfer (MMCT) from Fe(ii) of the ferrocene to Mo(iv). The relationship between electronic couplings and intramolecular MMCTs has already been observed in some transition-metal complexes bearing a 4-ferrocenylpyridine scaffold as a ligand.^18^ In fact, intramolecular MMCTs between the Fe atom in the ferrocene moiety and the Ru atom were observed in ruthenium complexes bearing 4-ferrocenylpyridines18a,b and/or 4′-ferrocenyl-2,2′:6′,2′′-terpyridine as ligands.18*c*

The CVs of the molybdenum–trichloride complexes bearing Et^Fc^ and Ph^Fc^ groups, **3c** and **3d**, showed similar two merged reversible waves. On the basis of the result of the CV of **3b**, we assigned the former waves (*E*_1/2_: +0.05 and +0.06 V) to Mo(iii/iv) and the latter (*E*_1/2_: +0.19 and +0.13 V) to Fe(ii/iii) in the CVs of **3c** and **3d** (Table 2). In both cases, oxidation of the Mo atom at first may occur in the anodic scan and then oxidation of the Fe atom of the ferrocene unit in the PNP-pincer ligand may proceed sequentially. The *E*_1/2_ values of Fe(ii/iii) of the ferrocene units shift by +0.22 and +0.09 V, respectively, upon coordination. These results indicate that the electronic communication is weakened by the introduction of ethylene or phenylene group into the linkage between the ferrocene and the pyridine of the PNP-pincer ligand. Similar phenomena have already been observed in a rhodium complex bearing 4-(4-ferrocenylphenyl)pyridine as a ligand17*c* and diruthenium complexes bearing a bridging dipyridine ligand.^19^ In the latter case, the peak to peak separation in the CVs of the dinuclear ruthenium complexes decreased with the increase of the metal separation of the two Ru centers in the complex.^19^

The CV of the molybdenum–trichloride complex bearing a Rc-substituted PNP-pincer ligand, **3e**, showed only one reversible wave assignable to Mo(iii/iv) in the electrochemical window, indicating that the Rc group in **3e** did not act as a redox active moiety, in contrast to the ferrocene units in **3b**, **3c** and **3d** (*vide supra*).

### 2.3. Evaluation of electronic interaction between Fe and Mo atoms in ammonia formation

**Scheme 2:**
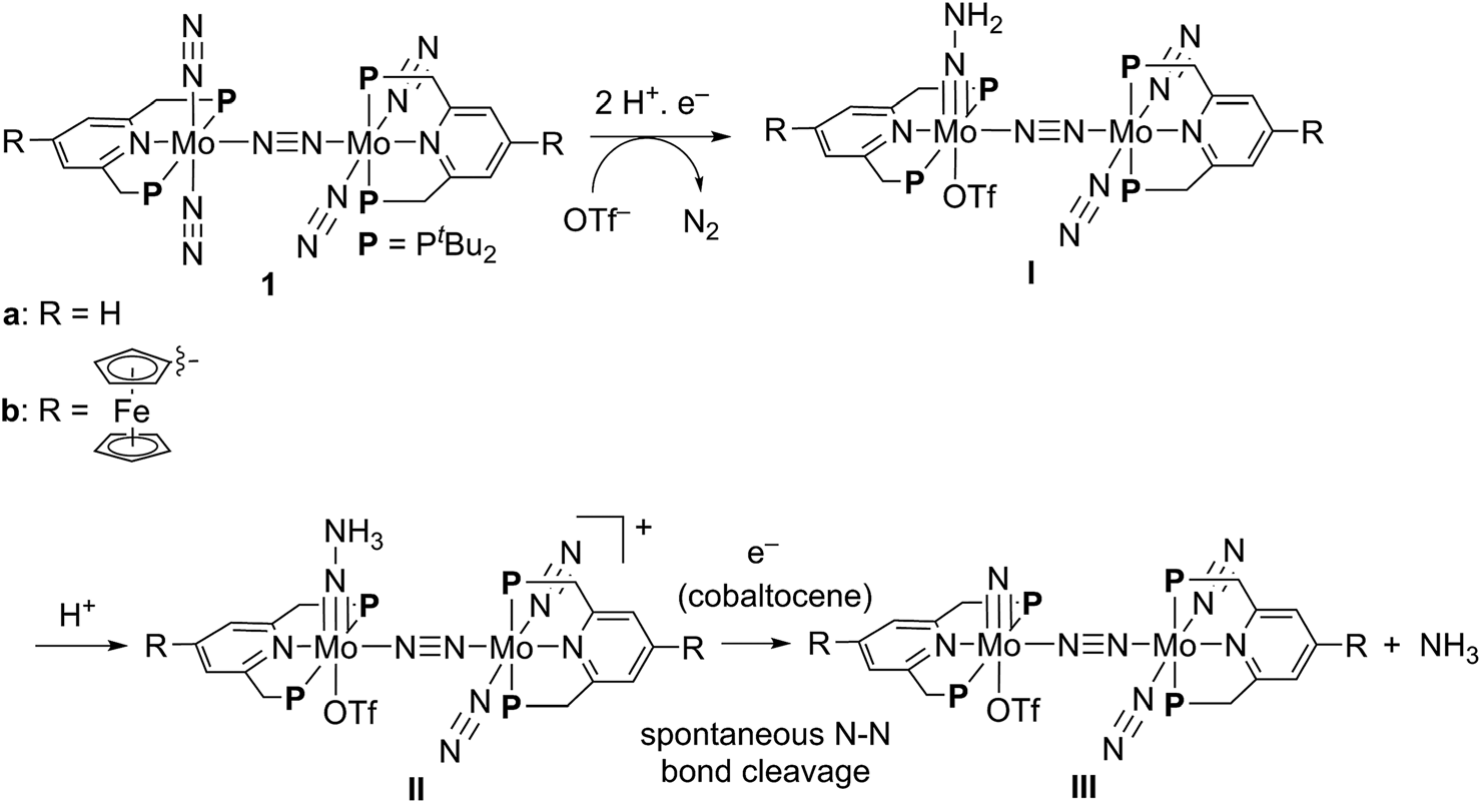
Reaction pathway from dinitrogen-bridged dimolybdenum–dinitrogen complex **1** into dinitrogen-bridged dimolybdenum–nitride complex **III***via* dinitrogen-bridged dimolybdenum–hydrazide and –hydrazidium complexes **I** and **II**.

**Fig. 6:**
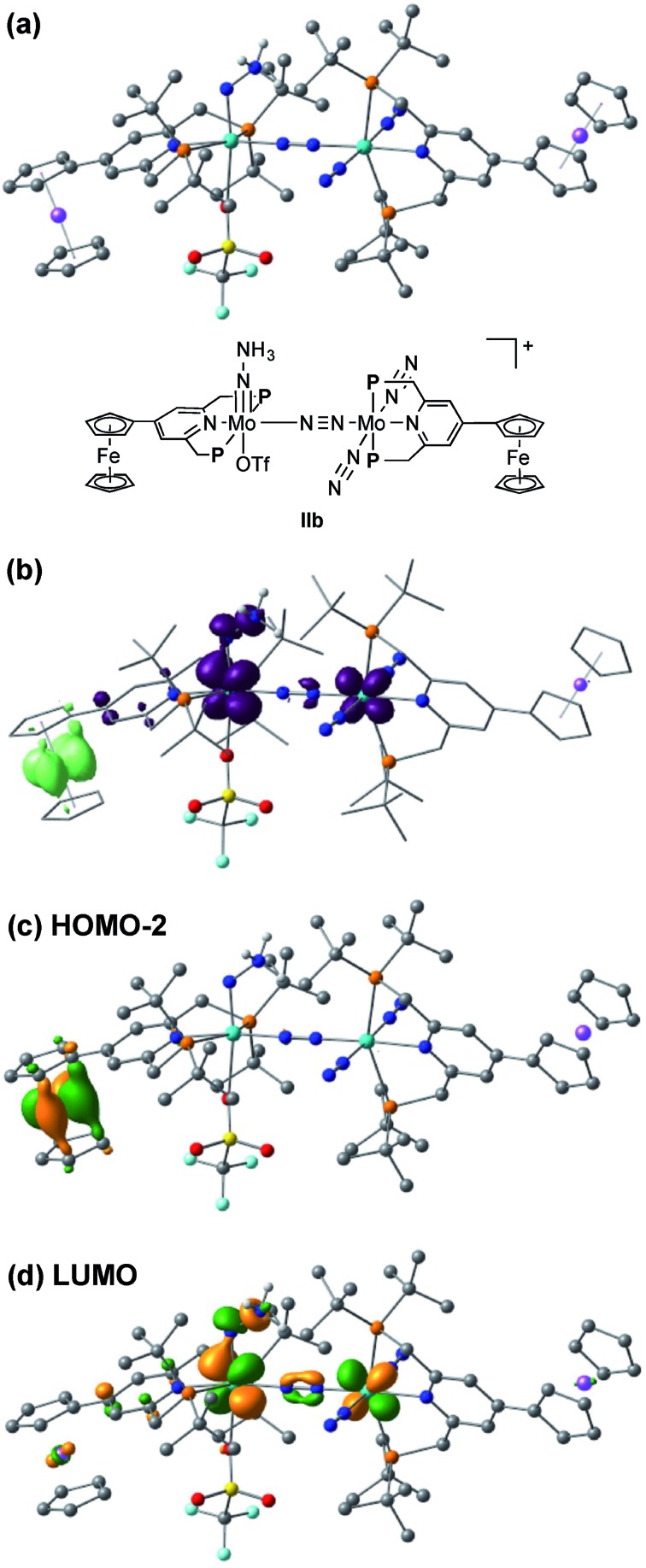
(a) Optimized structure of the ferrocene-substituted dinitrogen-bridged dimolybdenum–hydrazidium complex [Mo(4-Fc-PNP)(OTf)(NNH_3_)–N≡N–Mo(4-Fc-PNP)(N_2_)_2_]^+^ (**IIb**). (b) EDDM of the electron transition at 1174 nm based on the time-dependent BP86 result. (c) Molecular orbitals responsible for the transition at 1174 nm.

The electrochemical and theoretical studies revealed the presence of electronic interaction between the Mo and Fe atoms in the series of molybdenum–trichloride complexes bearing a ferrocene-substituted PNP-pincer ligand. Such interactions could enhance the catalytic ability of the dinitrogen-bridged dimolybdenum complexes *via* an electron transfer from the Fe atom in the ferrocene moiety to the Mo atom of the complexes bearing nitrogenous ligands that are transformed into ammonia. Among these molybdenum complexes proposed as reactive intermediates in the catalytic cycle in our previous study,^7^ we have found that a dinitrogen-bridged dimolybdenum–hydrazidium (–NNH_3_) complex met our hypothesis. In the reaction pathway starting from **1a**, dinitrogen-bridged dimolybdenum–hydrazide(2–) complex [Mo(PNP)(OTf)(NNH_2_)–N≡N–Mo(PNP)(N_2_)_2_] (**Ia**) is protonated to yield the corresponding dinitrogen-bridged dimolybdenum–hydrazidium complex [Mo(PNP)(OTf)(NNH_3_)–N≡N–Mo(PNP)(N_2_)_2_]^+^ (**IIa**), which is then reduced with cobaltocene to afford ammonia and the dinitrogen-bridged dimolybdenum–nitride complex [Mo(PNP)(OTf)(N)–N≡N–Mo(PNP)(N_2_)_2_] (**IIIa**) (Scheme 2). The protonation of **Ia** is slightly exothermic (Δ*E* = –1.6 kcal mol^–1^) with very low activation energy of 1.9 kcal mol^–1^, while the reduction of **IIa** followed by the N–N bond cleavage is extremely exothermic (Δ*E* = –156.1 kcal mol^–1^).^7^ We have now carried out DFT calculations to examine the impact of the introduction of ferrocene to the pyridine ring of the PNP-pincer ligand. Fig. 6(a) presents an optimized structure of the ferrocene-substituted dinitrogen-bridged dimolybdenum–hydrazidium complex [Mo(4-Fc-PNP)(OTf)(NNH_3_)–N≡N–Mo(4-Fc-PNP)(N_2_)_2_]^+^ (**IIb**). For electronic transitions of **IIb** calculated by the TD-BP86 method (Fig. 6(b)), a low-energy transition at 1174 nm is assignable to the electron transfer from the Fe atom of the ferrocene moiety to the Mo atom. Fig. 6(c) and (d) describe the EDDM of the transition at 1174 nm and molecular orbitals responsible for the transition (HOMO–2 and LUMO). This can be assigned to a MMCT transition from the Fe atom to the Mo atom. It should also be noted that the LUMO of **IIb** has antibonding character between the two N atoms of the NNH_3_ ligand. Thus, the N–N bond of the NNH_3_ ligand in **IIb** will be more activated, once an electron occupied the LUMO. As presented in our previous report, a neutral species formed by the reduction of the hydrazidium complex, **IIa**, was easily transformed to a pair of the nitride complex **IIIa** and ammonia *via* a spontaneous N–N bond cleavage.^7^ If the ferrocene-substituted PNP-pincer ligand enables the Mo center of the hydrazidium complex **IIb** to utilize an intramolecular MMCT, the formation of ammonia would be promoted by the reduction of the Mo–NNH_3_ moiety. As a result, we expect that the acceleration of the reduction step from the hydrazidium complex to the nitride complex provides an advantage for the catalytic reaction, because the protonation of the hydrazide complex into the hydrazidium complex is an isoenergetic process with a very small activation barrier, and the backward reaction (proton detachment) of the hydrazidium complex into the hydrazide complex is also likely to occur before the reduction of the hydrazidium complex with cobaltocene, producing the nitride complex and ammonia. At present, we can not exclude another possibility such as the acceleration of other steps and the stabilization of some reactive intermediates in the catalytic cycle by the introduction of the ferrocene moiety to the catalyst.

### 2.4. Catalytic formation of ammonia from molecular dinitrogen

#### 2.4.1. Catalytic reactions by using metallocene-substituted dinitrogen-bridged dimolybdenum–dinitrogen complexes

We carried out the catalytic reduction of molecular dinitrogen into ammonia using the complex **1** as a catalyst, according to the following procedure of the previous method.^9^ To a mixture of **1** and 2,6-lutidinium trifluoromethanesulfonate (288 equiv. to **1**) as a proton source in toluene was added a solution of cobaltocene (216 equiv. to **1**; CoCp_2_) as a reductant in toluene *via* a syringe pump at room temperature over a period of 1 h, and the resulting mixture was stirred at room temperature for another 19 h under 1 atm of dinitrogen. After the reaction, the formation of ammonia together with molecular dihydrogen was observed, the amounts of ammonia and molecular dihydrogen being determined by indophenol method^20^ and GC, respectively. Typical results are shown in Table 3. The yields of ammonia and molecular dihydrogen were estimated on the basis of cobaltocene. No formation of other products such as hydrazine was observed in all cases.

**Table 3:** Molybdenum-catalyzed reduction of molecular dinitrogen into ammonia under ambient reaction conditions^*a*^

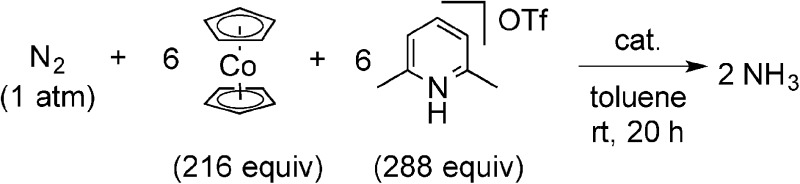						
Run	Cat.	R	NH_3_^*b*^ (equiv.)	NH_3_^*c*^ (%)	H_2_^*b*^ (equiv.)	H_2_^*c*^ (%)
1	**1b**	Fc	37^*f*^	51	36^*h*^	33
2^*d*^	**1a**	H	23	31	46	43
3^*e*^	**1a**	H	21^*g*^	29	44^*h*^	41
4	**1c**	Et^Fc^	30^*f*^	41	35^*f*^	32
5	**1d**	Ph^Fc^	10^*f*^	14	50^*g*^	46
6	**1e**	Rc	25^*g*^	35	38^*h*^	36
7^*d*^	**1h**	MeO	34	47	36	33

^*a*^To a mixture of the catalyst (**1**: 0.010 mmol) and [LutH]OTf (288 equiv. to the catalyst) as proton source in toluene (1.0 mL) was added a solution of CoCp_2_ (216 equiv. to catalyst) as a reductant in toluene (4.0 mL) at room temperature over a period of 1 h, followed by stirring the resulting mixture at room temperature for another 19 h under an atmospheric pressure of dinitrogen.

^*b*^Mol equiv. relative to the catalyst. Averages of multiple runs are shown.

^*c*^Yield based on CoCp_2_.

^*d*^ Ref. 9.

^*e*^FeCp_2_ (2 equiv. to **1a**) was added.

^*f*^Variation of ±2 equiv. between experiments.

^*g*^Variation of ±3 equiv. between experiments.

^*h*^Variation of ±1 equiv. between experiments.

The reaction by use of **1b** as a catalyst gave 37 equiv. of ammonia and 36 equiv. of molecular dihydrogen, respectively, based on the catalyst (Table 3, run 1).^21^,^22^ The amount of produced ammonia was higher than that obtained by using **1a** as a catalyst under the same reaction conditions (Table 3, run 2). For comparison, we carried out the catalytic reaction by using **1a** as a catalyst in the presence of 2 equiv. of ferrocene to **1a**, where 21 equiv. of ammonia were produced based on the catalyst (Table 3, run 3). These results indicated that the ferrocene moiety directly connected to the pyridine ring of the PNP-pincer ligand plays an important role to promote the catalytic reaction more efficiently. As shown in the previous section, the electronic density of the Mo atom of **1b** is almost the same with that of **1a** (Table 1). The presence of ferrocene at the PNP-pincer ligand in **1** did not affect the nature of the coordinated terminal dinitrogen ligand in **1**.^9^ Thus, the ferrocene moiety in **1b** may not act as an electron-donating group but rather as a redox active group for assisting to promote the reduction steps of the coordinated nitrogenous ligands on the Mo atom more smoothly.

**Fig. 7:**
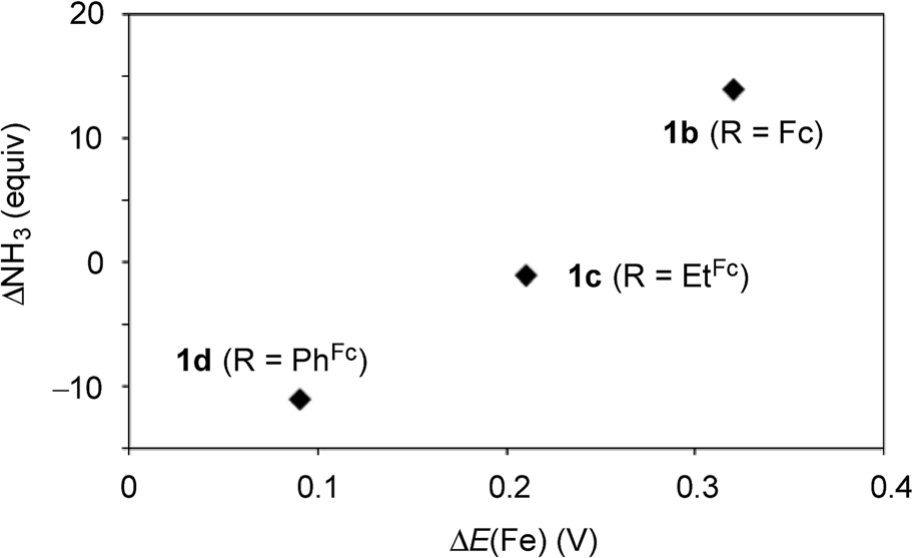
Relationship between the difference *E*_1/2_ value of Fe(ii/iii) of the ferrocene unit in the PNP-pincer ligand **2** on the coordination as **3** and the difference of the catalytic activity of **1**. See Table 4 in details.

Next, we carried out the catalytic ammonia formation by using other ferrocene-substituted dinitrogen-bridged dimolybdenum–dinitrogen complexes as catalysts. In the reactions using **1c** and **1d** as catalysts, lesser amounts of ammonia compared to that by use of **1b** was formed (30 equiv. and 10 equiv.^23^ of ammonia based on the catalysts, respectively) (Table 3, runs 4 and 5). The catalytic activity of **1** decreased with the decrease of the electrochemical interaction between the Mo atom and the Fe atom of the ferrocene unit in the PNP-pincer ligands (Table 4), and actually a good relationship between Δ*E*(Fe) [the difference of the Fe(ii/iii) potential (*E*_1/2_) between **2** and **3**] and ΔNH_3_ [the difference of the catalytic activity of **1**] was observed as shown in Fig. 7. This result also shows that the complex having a larger Δ*E*(Fe) value has a higher catalytic activity for ammonia formation.

**Table 4:** Difference of catalytic activity of dinitrogen-bridged dimolybdenum–dinitrogen complexes and difference of *E*_1/2_ value of Fe(ii/iii) couple upon coordination

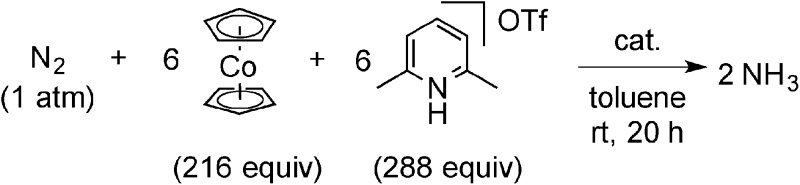								
Run	Complex bearing ferrocene moiety			Reference complex^*a*^			ΔNH_3_^*b*^ (equiv.)	Δ*E*(Fe)^*c*^/V
	Complex	R	NH_3_^*b*^ (equiv.)	Complex	R	NH_3_^*b*^ (equiv.)		
1	**1b**	Fc	37	**1a**	H	23	14^*d*^	0.32
2	**1c**	Et^Fc^	30	**1f**	Me	31	–1^*e*^	0.22
3	**1d**	Ph^Fc^	10	**1g**	Ph	21	–11^*f*^	0.09

^*a*^ Ref. 9.

^*b*^The amount of ammonia based on the catalyst.

^*c*^Difference of *E*_1/2_ value of the Fe(ii/iii) couple of the ferrocene moiety between the corresponding PNP-pincer ligand **2** and the molybdenum–trichloride complex **3**. See Table 2.

^*d*^Difference between the amount of ammonia catalyzed by **1b** and that by **1a**.

^*e*^Difference between the amount of ammonia catalyzed by **1c** and that by **1f**.

^*f*^Difference between the amount of ammonia catalyzed by **1d** and that by **1g**.

The reaction using the ruthenocene-substituted dinitrogen-bridged dimolybdenum–dinitrogen complex **1e** as a catalyst under the same reaction conditions gave only 25 equiv. of ammonia based on the catalyst (Table 3, run 6). The catalytic activity of **1e** was almost the same with that of **1a** and lower than that of **1b**. This result indicates that the ruthenocene moiety in **3e** did not act as a redox active group in the present reaction system.

#### 2.4.2. Nature of ferrocene-substituted dinitrogen-bridged dimolybdenum–dinitrogen complexes

**Scheme 3:**
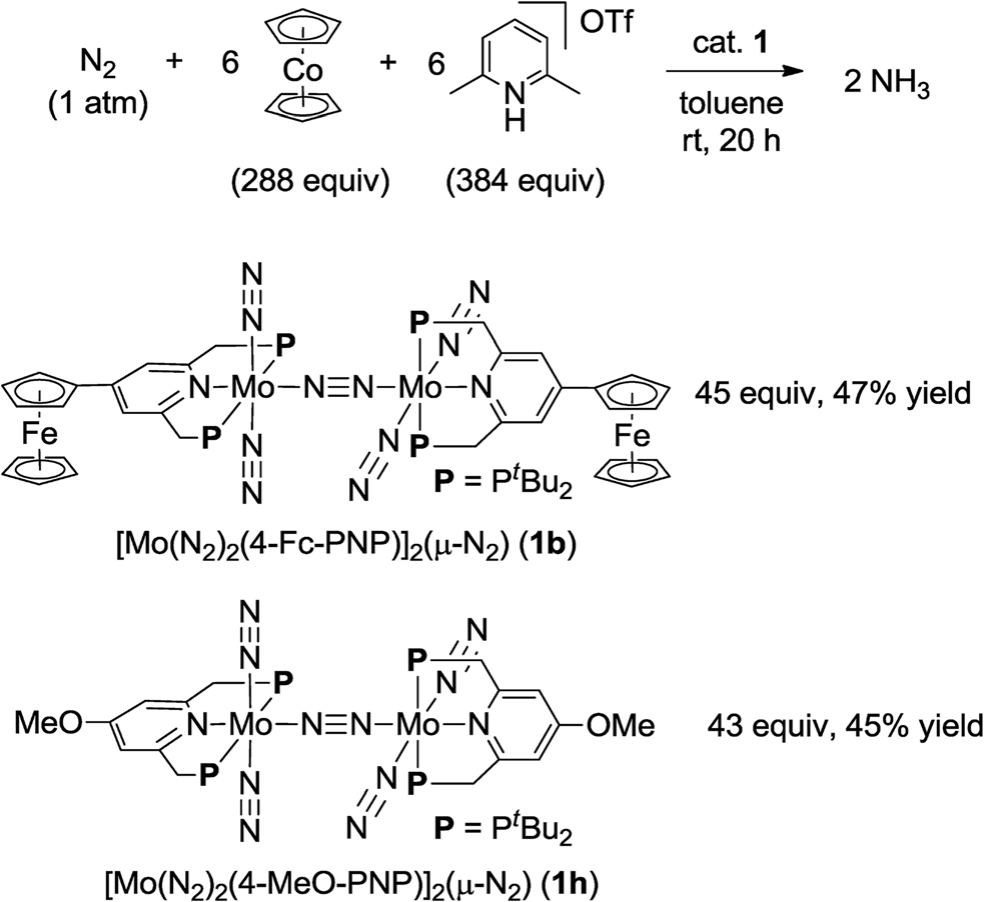
Catalytic formation of ammonia from molecular dinitrogen in the presence of **1b** and **1h** as catalysts by using larger amounts of cobaltocene and proton source.

We previously reported the nature of substituents at PNP-pincer ligands in dinitrogen-bridged dimolybdenum–dinitrogen complexes affects the catalytic activity for ammonia formation.^9^ Especially, the dinitrogen-bridged dimolybdenum–dinitrogen complex bearing 4-methoxy-substituted PNP-pincer ligands [Mo(N_2_)_2_(4-MeO-PNP)]_2_(μ-N_2_) (**1h**) worked as the most effective catalyst toward the catalytic ammonia formation under ambient reaction conditions.^9^ However, the formation of ammonia by using **1h** as a catalyst proceeded sluggishly. For comparison with the catalytic activity of **1b** toward ammonia formation, we previously carried out the catalytic reaction by using **1h** as a catalyst under the present reaction conditions, where 34 equiv. of ammonia were formed based on the catalyst (Table 3, run 7).^9^ In addition, when larger amounts of CoCp_2_ (288 equiv. to **1**) and [LutH]OTf (384 equiv. to **1**) were used as reductant and proton source under the same reaction conditions, 45 equiv. (**1b** as a catalyst) and 43 equiv. (**1h** as a catalyst) of ammonia were produced based on the catalysts, respectively (Scheme 3). This result indicated that the catalytic activity of **1b** is almost the same as that of **1h**, which was so far known to work as the most effective catalyst toward the formation of ammonia under ambient reaction conditions.^9^

**Fig. 8:**
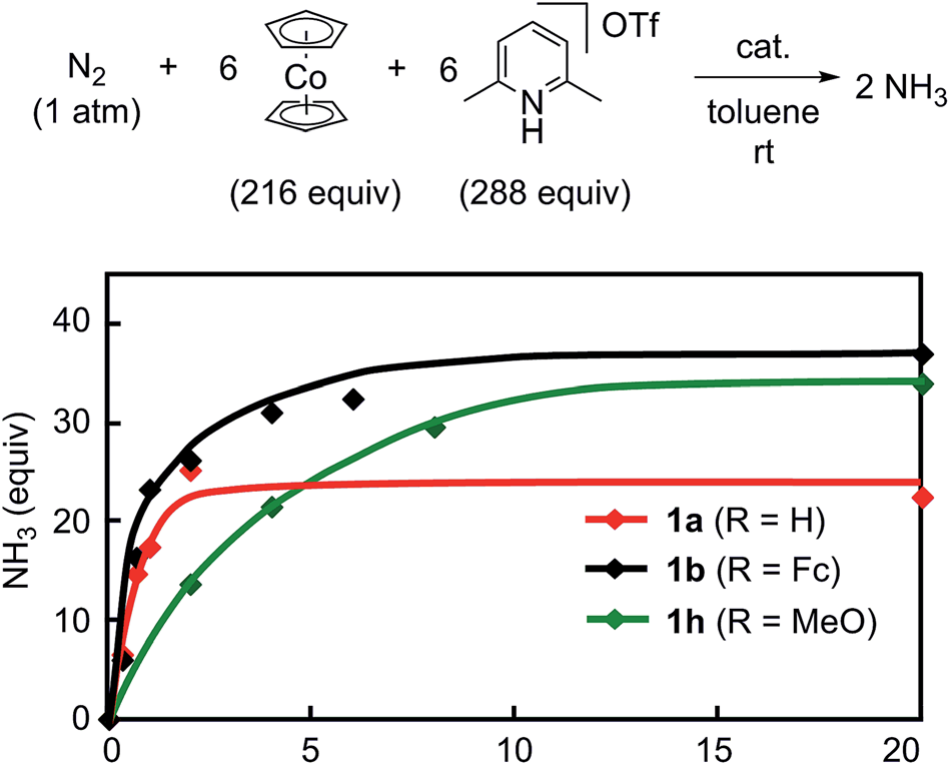
Time profiles of ammonia formation with **1a** (red), **1b** (black) and **1h** (green) as catalysts.

To obtain more information on the catalytic behavior of **1b**, we monitored the time profile of catalytic reactions by using **1b** as a catalyst. Typical results are shown in Fig. 8 together with the time profiles of catalytic reactions by using **1a** and **1h** as catalysts. The catalytic conversion of molecular dinitrogen into ammonia in the presence of a catalytic amount of **1b** proceeded more rapidly than that with **1a** and **1h**, and the turnover frequency (TOF) for ammonia, which was determined by mols of ammonia produced in initial 1 h per catalyst, by **1b** as a catalyst was 23 h^–1^. Typical results are shown in Table 5. On the other hand, the catalytic formation of ammonia in the presence of catalytic amounts of **1a** and **1h** proceeded more slowly than that of **1b** under the same reaction conditions.^9^ In fact, the TOFs for ammonia formation using **1a** and **1h** as catalysts, 17 h^–1^ and 7 h^–1^, respectively, were lower than that using **1b**.^9^ These results indicate that the ferrocene-substituted dinitrogen-bridged dimolybdenum–dinitrogen complex **1b** has the highest performance from the viewpoint of both the catalytic ability and the rate for ammonia formation.

**Table 5:** TOF and TON values for the formation of ammonia and molecular dihydrogen catalyzed by **1**^*a*^

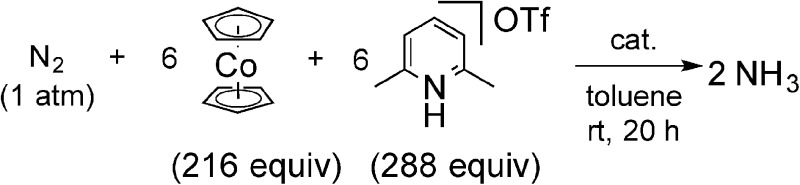					
Complex	R	TOF (NH_3_)/h^–1^	TOF (H_2_)/h^–1^	TON (NH_3_)	TON (H_2_)
**1b**	Fc	23^*b*^	17^*c*^	37^*c*^	36^*b*^
**1a**	H	17	23	23	46
**1h**	MeO	7	5	34	36

^*a*^TOF and TON are determined based on the catalyst. Average of multiple runs is shown. See Table 3 for reaction conditions. Values of **1a** and **1h** are from ref. 9.

^*b*^Variation of ±1 equiv. between experiments.

^*c*^Variation of ±2 equiv. between experiments.

**Fig. 9:**
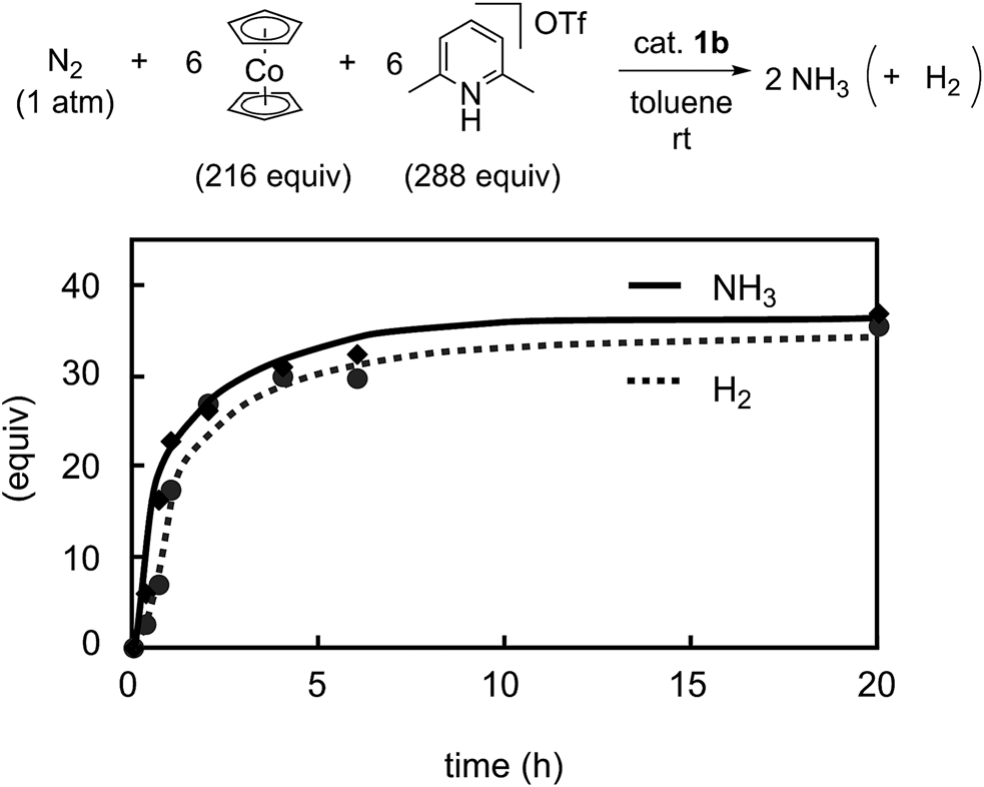
Time profiles of the formation of ammonia (solid line) and molecular dihydrogen (dashed line) with **1b** as a catalyst.

Previously we disclosed that the formation of ammonia and molecular dihydrogen is complementary in the present reaction system.^9^ To obtain more information on the catalytic activity of **1b** toward the formation of molecular dihydrogen, we monitored it in the catalytic reaction using **1b** as a catalyst. Typical results are shown in Fig. 9. The formation of molecular dihydrogen proceeded simultaneously with ammonia formation, where the TOF for molecular dihydrogen using **1b** as a catalyst is 17 h^–1^ (Table 5). As shown in the previous paper, the TOF for molecular dihydrogen using **1a** as a catalyst is 23 h^–1^.^9^ Thus, the TOF of **1b** for molecular dihydrogen is lower than that of **1a**. These results indicate that **1b** can promote the formation of ammonia and, on the other hand, suppress the formation of molecular dihydrogen.

**Fig. 10:**
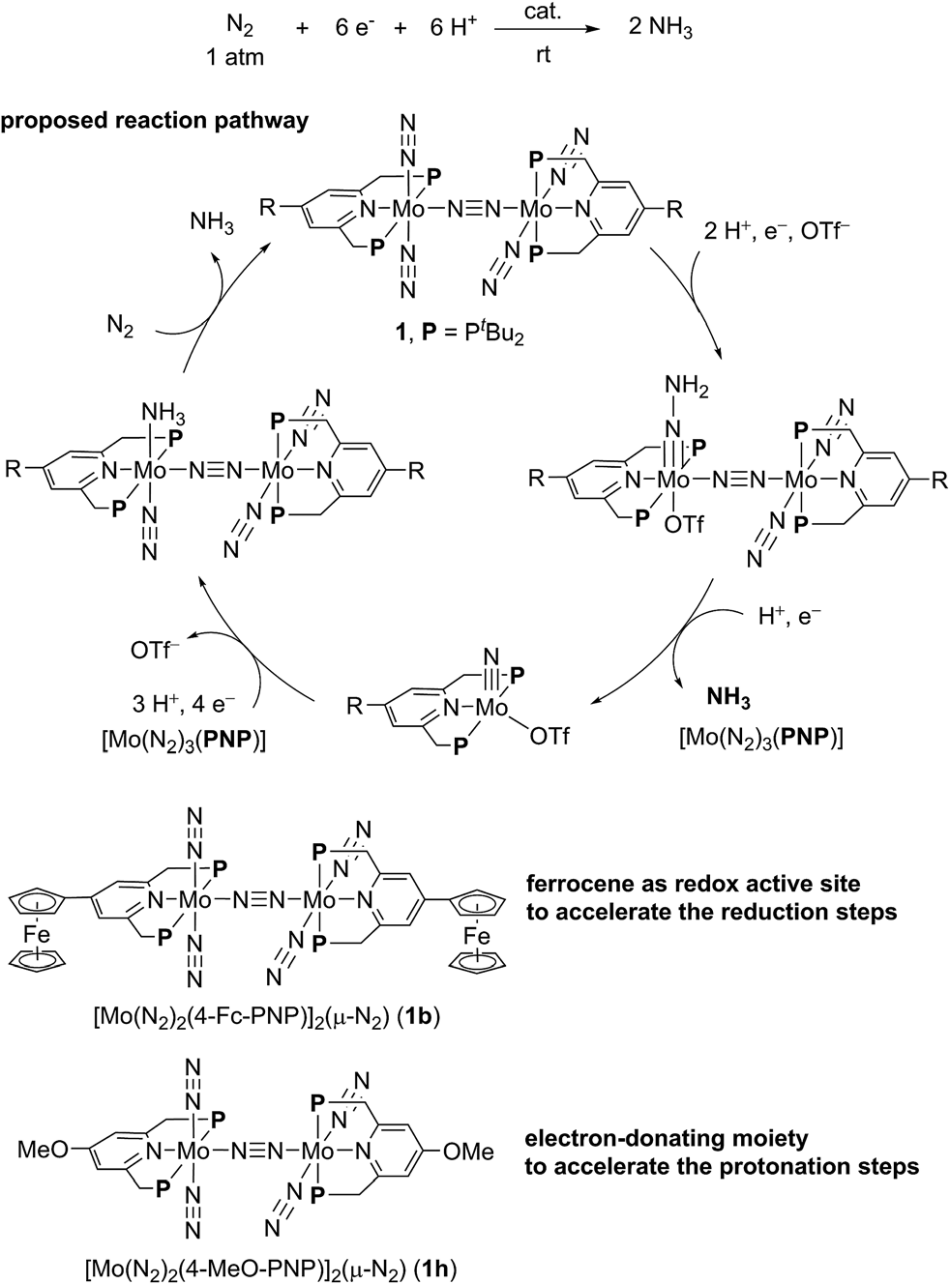
The role of substituent to the PNP-pincer ligand toward catalytic ammonia formation from molecular dinitrogen.

Based on the unique behavior and the DFT calculation result of **1b**, we consider that the presence of the ferrocene moiety directly connected to the pyridine ring of the PNP-pincer ligand in the catalyst increases the rate of the reduction steps of the coordinated nitrogenous ligand on the Mo atom. In this reaction system, the electronic interaction between the Fe atom of the ferrocene moiety and the Mo atom of the complex plays an important role to promote the catalytic formation of ammonia more smoothly, where the ferrocene moiety works as an intramolecular reductant, clarified by the reversible one-electron redox behavior, toward the high-oxidation state of the Mo complexes such as hydrazidium complexes. The observed effect of direct introduction of a ferrocene moiety to the pyridine ring of the PNP-pincer ligand is in sharp contrast to the effect observed by introduction of an electron-donating group such as methoxy group to the pyridine ring of the PNP-pincer ligand. In the latter case, the electron-donating group accelerates the protonation step of the coordinated nitrogenous ligands on the Mo atom of the complexes in the catalytic reaction (Fig. 10), while a ferrocene moiety increases the rate of the reduction step.^9^ At present, we can not exclude another possibility of the role of the ferrocene moiety in the catalyst and further work is necessary to clarify the exact role of the ferrocene moiety in the catalyst. However, the result described in the present paper provides the first successful example of the substantial improvement of the catalytic reactivity by the presence of the ferrocene moiety as a redox active site in the catalytic reduction of molecular dinitrogen into ammonia under ambient reaction conditions.^24^

The electron transfer from the Fe atom of the ferrocene moiety to the active site of the Mo atom in the complex plays an important role to promote catalytic ammonia formation effectively. As presented in the introduction part, a similar electron transfer may occur at the active site of nitrogenase enzyme in biological nitrogen fixation (Fig. 1). We believe that the mechanistic insight obtained in the present paper may provide valuable information to understand the mechanism of biological nitrogen fixation by nitrogenase.

## 3. Conclusion

We have prepared and characterized a series of dinitrogen-bridged dimolybdenum–dinitrogen complexes bearing metallocene-substituted PNP-pincer ligands spectroscopically and theoretically. The complex bearing a ferrocene group on the pyridine ring of the PNP-pincer ligand **1b** has been found to work as the most efficient catalyst toward the catalytic formation of ammonia from molecular dinitrogen, where up to 45 equiv. of ammonia were produced based on the catalyst (22 equiv. of ammonia based on each Mo atom of the catalyst). The catalytic activity of **1b** is almost the same with that of **1h**, which was previously reported to work as the most effective catalyst toward ammonia formation under ambient reaction conditions.^9^ Mechanistic study indicated that the presence of ferrocene as a redox active moiety in the PNP-pincer ligand increases the rates of the reduction step involved in the catalytic process of ammonia formation *via* an intramolecular electron transfer from the Fe atom of the ferrocene to the active site of the Mo atom in the complex. The present result is another useful innovation to the previous finding that the introduction of an electron-donating group to the pyridine ring of the PNP-pincer ligand accelerated the protonation step involved in the catalytic reaction.^9^ We believe that the result described in this article provides useful information to design a more effective catalyst toward the catalytic formation of ammonia from molecular dinitrogen under ambient reaction conditions. Further work is currently in progress to develop a more effective reaction system.^25^

## Supplementary Material

Supplementary information

Crystal structure data

Crystal structure data
