# Supplementary material for: Nitrogen fixation catalyzed by ferrocene-substituted dinitrogen-bridged dimolybdenum–dinitrogen complexes: unique behavior of ferrocene moiety as redox active site
Source: Chem Sci. 2015 Apr 20;6(7):3940–51. doi: 10.1039/c5sc00545k (PMC5707465; doi:10.1039/c5sc00545k)
Supplement: Supplementary file 1 [file SC-006-C5SC00545K-s001.pdf]

Electronic Supplementary Material (ESI) for Chemical Science.
This journal is © The Royal Society of Chemistry 2015

Supplementary Information

Nitrogen Fixation Catalyzed by Ferrocene-Substituted Dinitrogen-Bridged Dimolybdenum-Dinitrogen Complexes: Unique Behavior of Ferrocene Moiety as Redox Active Site

Shogo Kuriyama,† Kazuya Arashiba,† Kazunari Nakajima,† Hiromasa Tanaka,‡ Kazunari Yoshizawa,*,‡§ and Yoshiaki Nishibayashi*,†

†*Institute of Engineering Innovation, School of Engineering, The University of Tokyo, Yayoi, Bunkyo-ku, Tokyo 113-8656, Japan*

‡*Elements Strategy Initiative for Catalysts and Batteries (ESICB), Kyoto University, Nishikyo-ku, Kyoto 615-8520, Japan*

§*Institute for Materials Chemistry and Engineering and International Research Center for Molecular System, Kyushu University, Nishi-ku, Fukuoka 819-0395, Japan*

Experimental Methods.

^1H NMR (270 MHz), $^{31}P\{^1H\}$ NMR (109 MHz), $^{13}C\{^1H\}$ NMR (68 MHz), and $^{15}N\{^1H\}$ NMR (27 MHz) spectra were recorded on a JEOL Excalibur 270 spectrometer in suitable solvent, and spectra were referenced to residual solvent (^1H, $^{13}C\{^1H\}$) or external standard ($^{31}P\{^1H\}$: H_3PO_4, $^{15}N\{^1H\}$: CH_3NO_2). IR spectra were recorded on a JASCO FT/IR 4100 Fourier Transform infrared spectrometer. Raman spectra were recorded on a JASCO NRS-2000 or a Renishaw inVia Reflex Raman microscope. Absorption spectra were recorded on a Shimadzu MultiSpec-1500. Evolved dihydrogen was quantified by a gas chromatography using a Shimadzu GC-8A with a TCD detector and a SHINCARBON ST (6 m × 3 mm). Elemental analyses were performed at Microanalytical Center of The University of Tokyo. Mass spectra were measured on a JEOL JMS-700 mass spectrometer.

All manipulations were carried out under an atmosphere of nitrogen by using standard Schlenk techniques or glovebox techniques unless otherwise stated. Toluene was distilled from dark-blue Na/benzophenone ketyl solution and degassed, and stored over molecular sieves 4A in a nitrogen-filled glove box. Other solvents were dried by general methods, and degassed before use. $CoCp_2$ (Aldrich) was sublimed before use. 4-Bromo-2,6-dimethylpyridine,[S1] FcBpin (Fc = ferrocenyl, pin = pinacolate),[S2] RcBpin (Rc = ruthenocenyl),[S3] 4-ferrocenylbromobenzene,[S4] 2,6-bis((di-*t*-butylphosphinothioyl)methyl)-4-(4,4,5,5-tetramethyl-1,3,2-dioxaborolan-2-yl)pyridine,[S5] $MoCl_3(thf)_3$,[S6] $MoCl_3$(**PNP**) (**PNP** = 2,6-bis(di-*t*-butylphosphino)methylpyridine),[S7] $[Mo(N_2)_2$(**PNP**)$]_2(\mu\text{-}N_2)$ (**1a**),[S7] [LutH]OTf (OTf = trifluoromethanesulfonate, Lut = 2,6-lutidine),[S7] $[NBu_4]BAr^F_4$ (Ar^F = 3,5-$(CF_3)_2C_6H_3$),[S8] and $CrCp^*_2$ (Cp* = pentamethylcyclopentadienyl),[S9] were prepared according to the literature methods. [PicH]OTf (Pic = 2-picoline) and [ColH]OTf (Col = 2,4,6-collidine) were prepared in a similar method to [LutH]OTf.[S7] All the other reagents were commercially available.

Preparation of 4-ferrocenyl-2,6-dimethylpyridine (4b).

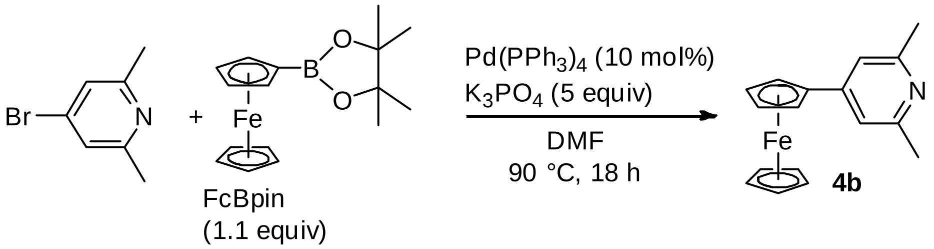

A suspension of 4-bromo-2,6-dimethylpyridine (552 mg, 2.97 mmol), FcBpin (1.03 g, 3.31 mmol), $Pd(PPh_3)_4$ (342 mg, 0.296 mmol), and K_3PO_4 (3.18 g, 15.0 mmol) in DMF (20 mL) was stirred at 90 ºC for 18 h. After the reaction mixture was cooled to room temperature, water was added. The resulting mixture was extracted with Et_2O, and the extracts were washed with water and then brine and dried over anhydrous $MgSO_4$. After filtration, the solvent was evaporated and the residue was purified by column chromatography on silica gel (hexane/AcOEt 2/1) to give **4b** as an orange solid (616 mg, 2.12 mmol, 71%). ^{1}H NMR (C_6D_6) δ 6.95 (s, Py-*H*, 2H), 4.49 (t, *J* = 1.9 Hz, Cp-*H*, 2H), 4.41 (t, *J* = 1.9 Hz, Cp-*H*, 2H), 3.86 (s, Cp-*H*, 5H), 2.53 (s, Py-*CH*$_3$, 6H). ^{13}C{^{1}H} NMR (C_6D_6) δ 158.0 (s, Ar), 148.6 (s, Ar), 117.2 (s, Ar), 82.4 (s, Cp), 70.1 (s, Cp), 69.9 (s, Cp), 67.1 (s, Cp), 24.7 (s, *C*H$_3$). Anal. Calcd. for $C_{17}H_{17}FeN$: C, 70.12; H, 5.88; N, 4.81. Found: C, 70.12; H, 6.00; N, 4.88.

Preparation of 4-(2-ferrocenylethenyl)-2,6-dimethylpyridine (**5**).

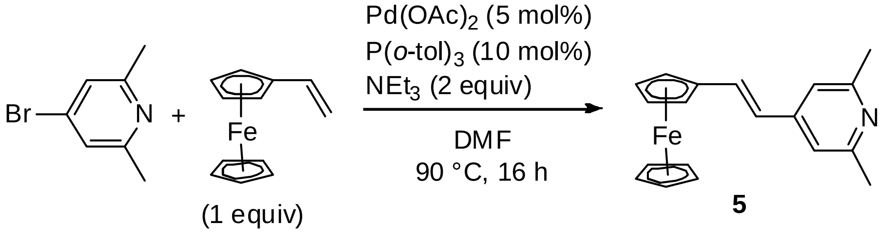

A solution of 4-bromo-2,6-dimethylpyridine (358 mg, 1.92 mmol), vinylferrocene (403 mg, 1.90 mmol), $Pd(OAc)_2$ (21 mg, 0.095 mmol), P(*o*-tol)$_3$ (58 mg, 0.19 mmol), and triethylamine (0.540 mL, 3.87 mmol) in DMF (9 mL) was stirred at 90 ºC for 16 h. After the reaction mixture was cooled to room temperature, water was added. The resulting mixture was extracted with Et_2O, and the extracts were washed with water and dried over anhydrous $MgSO_4$. After filtration, the solvent was evaporated and the residue was purified by column chromatography on silica gel (hexane/AcOEt 2/1) to give **5** as a reddish orange solid (483 mg, 1.52 mmol, 80%). ^{1}H NMR

($CDCl_3$) δ 7.09 (d, *J* = 15.9 Hz, vinyl-*H*, 1H), 6.98 (s, Py-*H*, 2H), 6.55 (d, *J* = 15.9 Hz, vinyl-*H*, 1H), 4.48 (t, *J* = 1.9 Hz, Cp-*H,* 2H), 4.34 (t, *J* = 1.9 Hz, Cp-*H,* 2H), 4.15 (s, Cp-*H*, 5H), 2.54 (s, Py-CH_3, 6H). $^{13}C\{^1H\}$ NMR ($CDCl_3$) δ 157.9 (s, Ar), 145.6 (s, Ar), 131.6 (s, vinyl), 123.5 (s, vinyl), 116.9 (s, Ar), 81.9 (s, Cp), 69.6 (s, Cp), 69.3 (s, Cp), 67.3 (s, Cp), 24.4 (s, CH_3). HRMS (FAB) Calcd. for $C_{19}H_{19}NFe$ [M]: 317.0867. Found: 317.0851.

Preparation of 4-(2-ferrocenylethyl)-2,6-dimethylpyridine (4c).

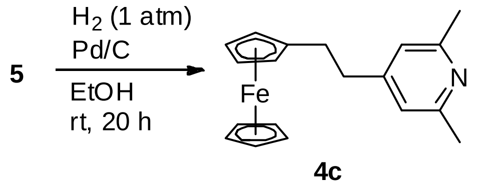

A suspension of **5** (692 mg, 2.18 mmol) and Pd/C (10% Pd, 50 mg) in EtOH (35 mL) was stirred at room temperature for 20 h under H_2 (1 atm). After filteration, the solvent was evaporated and the residue was purified by column chromatography on silica gel (hexane/AcOEt 3/1) to give **4c** as an orange oil (384 mg, 1.20 mmol, 55%). ^1H NMR (C_6D_6) δ 6.55 (s, Py-*H*, 2H), 4.00 (s, Cp-*H*, 5H), 3.96 (t, *J* = 1.6 Hz, Cp-*H*, 2H), 3.92 (t, *J* = 1.6 Hz, Cp-*H*, 2H), 2.58-2.40 (m, CH_2, 4H), 2.50 (s, Py-CH_3, 6H). $^{13}C\{^1H\}$ NMR (C_6D_6) δ 157.9 (s, Ar), 150.9 (s, Ar), 120.2 (s, Ar), 88.3 (s, Cp), 68.9 (s, Cp), 68.3 (s, Cp), 67.7 (s, Cp), 37.2 (s, CH_2), 31.0 (s, CH_2), 24.5 (s, CH_3). HRMS (FAB) Calcd. for $C_{19}H_{21}FeN$ [M]: 319.1023. Found: 319.1015.

Preparation of 4-ruthenocenyl-2,6-dimethylpyridine (4e).

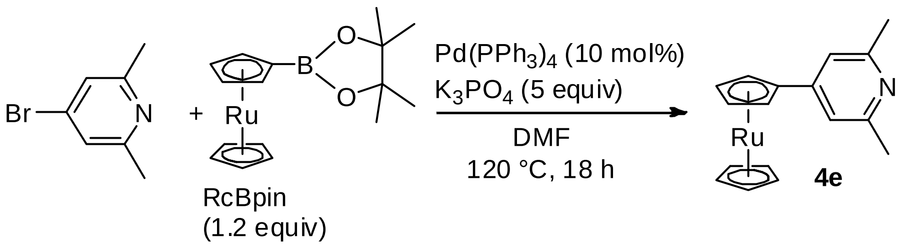

A suspension of 4-bromo-2,6-dimethylpyridine (542 mg, 2.92 mmol), RcBpin (1.40 g, 3.70 mmol), $Pd(PPh_3)_4$ (346 mg, 0.300 mmol), and K_3PO_4 (3.18 g, 15.0 mmol) in DMF (30 mL) was stirred at 120 ºC for 18 h. After the reaction mixture was cooled to room temperature, water was added and the mixture was filtered. The resulting mixture was extracted with Et_2O and the extracts were dried over anhydrous $MgSO_4$. After filtration, the solvent was evaporated and the

residue was purified by column chromatography on silica gel (hexane/AcOEt 2/1) to give **4e** as a yellow solid (681 mg, 2.02 mmol, 69%). ^{1}H NMR ($CDCl_3$) δ 6.93 (s, Py-*H*, 2H), 5.06 (t, *J* = 1.8 Hz, Cp-*H,* 2H), 4.70 (t, *J* = 1.8 Hz, Cp-*H,* 2H), 4.46 (s, Cp-*H*, 5H), 2.48 (s, Py-CH_3, 6H). ^{13}C{^{1}H} NMR ($CDCl_3$) δ 157.3 (s, Ar), 148.0 (s, Ar), 117.4 (s, Ar), 86.2 (s, Cp), 71.8 (s, Cp), 71.6 (s, Cp), 69.3 (s, Cp), 24.3 (s, CH_3). Anal. Calcd. for $C_{17}H_{17}RuN$: C, 60.70; H, 5.09; N, 4.16. Found: C, 60.73; H, 5.04; N, 3.88.

Preparation of 2,6-bis((di-*t*-butylphosphinothioyl)methyl)-4-(4-ferrocenylphenyl)pyridine (6).

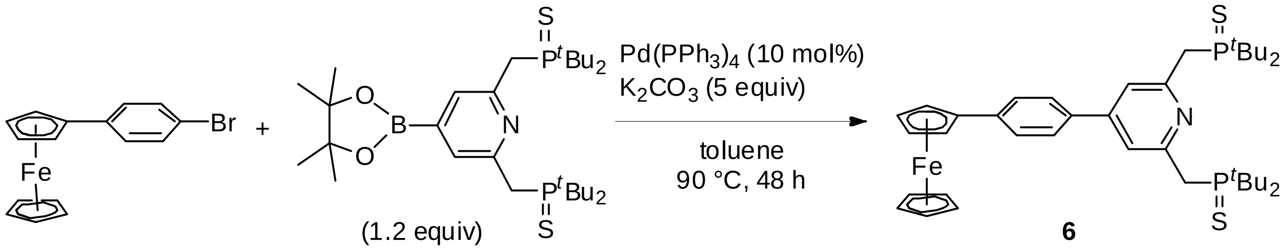

A suspension of 4-ferrocenylbromobenzene (409 mg, 1.20 mmol), 2,6-bis((di-*t*-butylphosphinothioyl)methyl)-4-(4,4,5,5-tetramethyl-1,3,2-dioxaborolan-2-yl)pyridine (843 mg, 1.44 mmol), $Pd(PPh_3)_4$ (138 mg, 0.119 mmol), and K_2CO_3 (823 mg, 5.95 mmol) in toluene (10 mL) was stirred at 90 ºC for 48 h. After the reaction mixture was cooled to room temperature, saturated aqueous $NaHCO_3$ solution was added. The resulting mixture was extracted with CH_2Cl_2, and the extracts were dried over anhydrous $MgSO_4$. After filtration, the solvent was evaporated and the residue was purified by column chromatography on silica gel (hexane/AcOEt 2/1) to give an orange solid. After recrystallization from boiling toluene, **6** was obtained as orange crystals (456 mg, 0.634 mmol, 53%). ^{1}H NMR ($CDCl_3$) δ 8.09 (s, Py-*H*, 2H), 7.75 (d, *J* = 8.4 Hz, Ar-*H,* 2H), 7.56 (d, *J* = 8.4 Hz, Ar-*H*, 2H), 4.70 (t, *J* = 1.6 Hz, Cp-*H*, 2H), 4.34 (t, *J* = 1.6 Hz, Cp-*H*, 2H), 4.06 (s, Cp-*H*, 5H), 3.54 (d, $J_{P\text{-}H}$ = 11.9 Hz, C*H*$_2$P^{t}Bu$_2$, 4H), 1.35 (d, $J_{P\text{-}H}$ = 14.9 Hz, CH$_2$P^t*Bu*$_2$, 36H). ^{31}P{^{1}H} NMR ($CDCl_3$) δ 76.6 (s). ^{13}C{^{1}H} NMR ($CDCl_3$) δ 153.6 (d, $J_{P\text{-}C}$ = 6.6 Hz, Ar), 146.9 (s, Ar), 140.4 (s, Ar), 135.2 (s, Ar), 127.4 (s, Ar), 126.5 (s, Ar), 121.6 (s, Ar), 84.5 (s, Cp), 69.7 (s, Cp), 69.2 (s, Cp), 66.6 (s, Cp), 38.5 (d, $J_{P\text{-}C}$ = 40.1 Hz, P*C*H$_2$), 34.1 (d, $J_{P\text{-}C}$ = 35.1 Hz, P*C*(CH$_3$)$_3$), 27.9 (d, $J_{P\text{-}C}$ = 1.1 Hz, PC(*C*H$_3$)$_3$). Anal. Calcd. for $C_{39}H_{55}FeNP_2S_2$: C, 65.08; H, 7.70; N, 1.95. Found: C, 65.02; H, 7.81; N, 1.84.

Preparation of 4-R-PNP (R = Fc (2b), R = Rc (2e)).

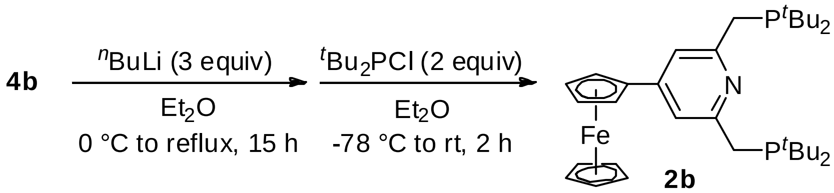

2b

PNP ligands (**2b** and **2e**) were prepared from a similar method to that of **PNP**.[S10] A typical procedure for the preparation of **2b** is described below.

To a solution of **4b** (406 mg, 1.40 mmol) in Et_2O (7 mL) was added dropwise ^{n}BuLi (1.59 M in hexane, 2.65 mL, 4.21 mmol) at 0 ºC. The reaction mixture was heated to reflux temperature for 15 h and then cooled to −78 ºC. ^{t}Bu$_2$PCl (0.500 mL, 2.63 mmol) was added dropwise, and then the reaction mixture was stirred at room temperature for 2 h. The reaction mixture was quenched with degassed water and extracted with Et_2O. The extracts were dried over anhydrous $MgSO_4$, and the solvent was removed and dried *in vacuo*. Recrystallization from Et_2O at −40 ºC afforded **2b** as an orange solid, which was collected by filtration and dried *in vacuo* (407 mg, 0.702 mmol, 53%). ^{1}H NMR (C_6D_6) δ 7.61 (s, Py-*H*, 2H), 4.64 (t, *J* = 1.9 Hz, Cp-*H*, 2H), 4.10 (t, *J* = 1.9 Hz, Cp-*H*, 2H), 3.97 (s, Cp-*H*, 5H), 3.19 (d, $J_{P\text{-}H}$ = 2.7 Hz, C*H*$_2$P^{t}Bu$_2$, 4H), 1.17 (d, $J_{P\text{-}H}$ = 10.8 Hz, CH$_2$P^t*Bu*$_2$, 36H). ^{31}P{^{1}H} NMR (C_6D_6) δ 34.2 (s). ^{13}C{^{1}H} NMR (C_6D_6) δ 161.4 (d, $J_{P\text{-}C}$ = 13.9 Hz, Ar), 148.3 (s, Ar), 118.3 (dd, $J_{P\text{-}C}$ = 10.6 Hz and $J_{P\text{-}C}$ = 1.4 Hz, Ar), 82.6 (s, Cp), 70.2 (s, Cp), 70.0 (s, Cp), 67.4 (s, Cp), 32.3 (d, $J_{P\text{-}C}$ = 26.0 Hz, P*C*(CH$_3$)$_3$), 31.9 (d, $J_{P\text{-}C}$ = 24.0 Hz, P*C*H$_2$), 30.0 (d, $J_{P\text{-}C}$ = 14.0 Hz, PC(*C*H$_3$)$_3$). Anal. Calcd. for $C_{33}H_{51}FeNP_2$: C, 68.39; H, 8.87; N, 2.42. Found: C, 68.57; H, 9.08; N, 2.71.

4-Rc-PNP (2e).

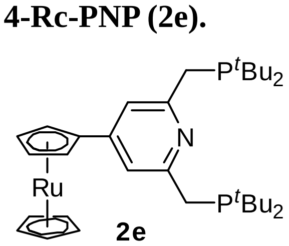

2e

2e: 48% Yield. A yellow solid. ^{1}H NMR (C_6D_6) δ 7.53 (s, Py-*H*, 2H), 5.08 (t, *J* = 1.8 Hz, Cp-*H*, 2H), 4.52 (t, *J* = 1.8 Hz, Cp-*H*, 2H), 4.44 (s, Cp-*H*, 5H), 3.13 (d, $J_{P\text{-}H}$ = 2.4 Hz, C*H*$_2$P^{t}Bu$_2$, 4H), 1.14 (d, $J_{P\text{-}H}$ = 10.5 Hz, CH$_2$P^t*Bu*$_2$, 36H). ^{31}P{^{1}H} NMR (C_6D_6) δ 34.1 (s). ^{13}C{^{1}H} NMR

(C_6D_6) δ 161.3 (d, $J_{P\text{-}C}$ = 14.0 Hz, Ar), 147.3 (s, Ar), 118.6 (dd, $J_{P\text{-}C}$ = 10.6 Hz and $J_{P\text{-}C}$ = 1.7 Hz, Ar), 87.5 (s, Cp), 72.1 (s, Cp), 71.8 (s, Cp), 70.0 (s, Cp), 32.3 (d, $J_{P\text{-}C}$ = 25.6 Hz, P*C*(CH_3)$_3$), 31.9 (d, $J_{P\text{-}C}$ = 24.0 Hz, P*C*H_2), 30.0 (d, $J_{P\text{-}C}$ = 14.0 Hz, PC(*C*H_3)$_3$). Anal. Calcd. for $C_{33}H_{51}NP_2Ru$: C, 63.44; H, 8.23; N, 2.24. Found: C, 63.35; H, 8.23; N, 2.22.

Preparation of 4-Et^{Fc}-PNP (2c).

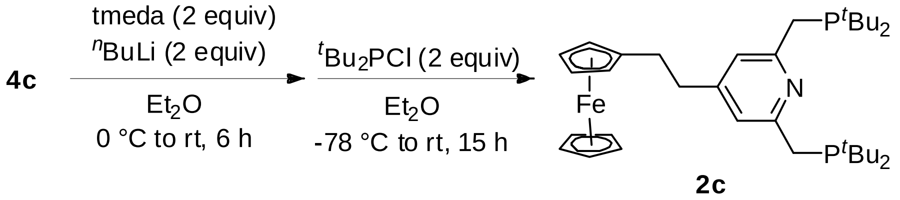

To a solution of **4c** (631 mg, 1.98 mmol) and tmeda (0.650 mL, 4.34 mmol) in Et_2O (6 mL) was added dropwise ^{n}BuLi (1.65 M in hexane, 2.60 mL, 4.29 mmol) at 0 ºC. The reaction mixture was stirred at 0 ºC for 4 h and then cooled to −78 ºC. ^{t}Bu$_2$PCl (0.760 mL, 4.00 mmol) was added dropwise, and then the reaction mixture was stirred at room temperature for 15 h. The reaction mixture was quenched with degassed water and extracted with Et_2O. The extracts were dried over anhydrous $MgSO_4$. After filtration, the solvent was removed and dried *in vacuo*. Recrystallization from Et_2O/hexane at −40 ºC afforded **2c** as an orange solid, which was collected by filtration and dried *in vacuo* (573 mg, 0.944 mmol, 48%). ^{1}H NMR (C_6D_6) δ 7.29 (s, Py-*H*, 2H), 3.96 (s, Cp-*H*, 5H), 3.92 (t, *J* = 1.8 Hz, Cp-*H*, 2H), 3.87 (t, *J* = 1.8 Hz, Cp-*H*, 2H), 3.16 (d, $J_{P\text{-}H}$ = 1.9 Hz, C*H*$_2$P^{t}Bu$_2$, 4H), 2.68-2.50 (m, *CH*$_2$, 4H), 1.18 (d, $J_{P\text{-}H}$ = 10.5 Hz, CH$_2$P^t*Bu*$_2$, 36H). ^{31}P{^{1}H} NMR (C_6D_6) δ 33.9 (s). ^{13}C{^{1}H} NMR (C_6D_6) δ 161.5 (d, $J_{P\text{-}C}$ = 14.5 Hz, Ar), 150.8 (s, Ar), 121.3 (dd, $J_{P\text{-}C}$ = 9.8 Hz and $J_{P\text{-}C}$ = 1.7 Hz, Ar), 88.2 (s, Cp), 68.8 (s, Cp), 68.4 (s, Cp), 67.6 (s, Cp), 37.3 (s, *C*H$_2$), 32.3 (d, $J_{P\text{-}C}$ = 25.6 Hz, P*C*(CH_3)$_3$), 31.9 (d, $J_{P\text{-}C}$ = 23.9 Hz, P*C*H_2), 30.9 (s, *C*H$_2$), 30.0 (d, $J_{P\text{-}C}$ = 14.0 Hz, PC(*C*H_3)$_3$). Anal. Calcd. for $C_{35}H_{55}FeNP_2$: C, 69.18; H, 9.12; N, 2.31. Found: C, 69.03; H, 9.10; N, 2.67.

Preparation of 4-Ph^{Fc}-PNP (2d).

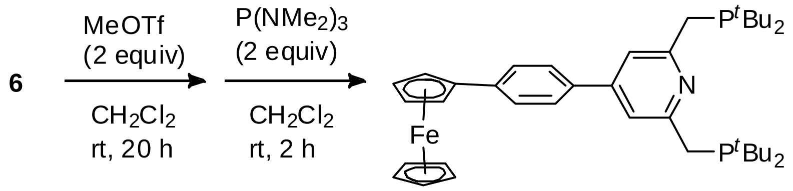

2d

To a solution of **6** (1.01 g, 1.40 mmol) in CH_2Cl_2 (25 mL) at room temperature was added dropwise MeOTf (0.310 mL, 2.83 mmol). The reaction mixture was stirred at room temperature for 20 h and then $P(NMe_2)_3$ (0.510 mL, 2.81 mmol) was added dropwise. After stirring at room temperature for 2 h, the solvent was concentrated under reduced pressure. The residue was extracted with Et_2O, and removal of the solvent gave **2d** as an orange solid (778 mg, 1.19 mmol, 85%). This sample was used for subsequent reaction without further purification. ^{1}H NMR (C_6D_6) δ 7.82 (s, Py-*H*, 2H), 7.64 (d, J = 8.4 Hz, Ar-*H*, 2H), 7.36 (d, J = 8.4 Hz, Ar-*H*, 2H), 4.51 (t, J = 1.9 Hz, Cp-*H*, 2H), 4.16 (t, J = 1.9 Hz, Cp-*H*, 2H), 3.92 (s, Cp-*H*, 5H), 3.24 (d, $J_{P\text{-}H}$ = 2.7 Hz, C*H*$_2$P^{t}Bu$_2$, 4H), 1.18 (d, $J_{P\text{-}H}$ = 10.8 Hz, CH$_2$P^t*Bu*$_2$, 36H). ^{31}P{^{1}H} NMR (C_6D_6) δ 34.5 (s). ^{13}C{^{1}H} NMR (C_6D_6) δ 162.3 (d, $J_{P\text{-}C}$ = 14.5 Hz, Ar), 148.3 (s, Ar), 140.4 (s, Ar), 136.8 (s, Ar), 127.4 (s, Ar), 126.9 (s, Ar), 118.8 (dd, $J_{P\text{-}C}$ = 10.6 Hz and 1.7 Hz, Ar), 85.0 (s, Cp), 70.0 (s, Cp), 69.5 (s, Cp), 66.9 (s, Cp), 32.5 (d, $J_{P\text{-}C}$ = 25.6 Hz, P*C*(CH$_3$)$_3$), 31.9 (d, $J_{P\text{-}C}$ = 24.0 Hz, P*C*H$_2$), 30.0 (d, $J_{P\text{-}C}$ = 14.0 Hz, PC(*C*H$_3$)$_3$). HRMS (FAB) Calcd. for $C_{39}H_{56}FeNP_2$ [M+H]: 656.3237. Found: 656.3255.

Preparation of [MoCl$_3$(4-R-PNP)] (3b - 3f).

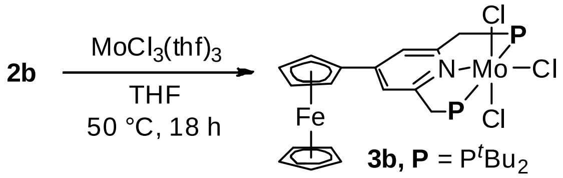

A typical procedure for the preparation of [MoCl$_3$(**2b**)] (**3b**) is described below. A mixture of **3b** (290 mg, 0.500 mmol) and MoCl$_3$(thf)$_3$ (193 mg, 0.460 mmol) in THF (10 mL) was stirred 50 ˚C for 18 h. The resultant suspension was concentrated under reduced pressure. The residue was recrystallized from CH_2Cl_2–hexane (4 mL/20 mL) to give orange crystals of **3b**·C_6H_{14}, which were collected by filtration and washed with Et_2O (3 mL, 3 times) and dried *in vacuo* to afford **3b** as a

red solid (322 mg, 0.412 mmol, 90% yield). Anal. Calcd. for $C_{33}H_{51}Cl_3FeMoNP_2$: C, 50.69; H, 6.57; N, 1.79. Found: C, 50.68; H, 6.57; N, 1.95.

[MoCl$_3$(2c)] (3c).

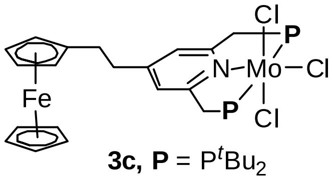

3c, P = P^{t}Bu$_2$

3c: 92% Yield. An orange solid. Anal. Calcd. for $C_{35}H_{55}Cl_3FeMoNP_2$: C, 51.90; H, 6.84; N, 1.73. Found: C, 51.07; H, 6.79; N, 1.38. No satisfactory of the elemental analysis is due to amounts of the solvent molecules, which depended on the isolation procedure of the complex.

[**MoCl$_3$(2d)] (3d).**

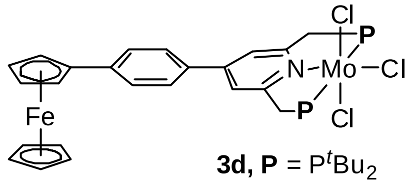

3d, P = P^{t}Bu$_2$

3d: 66% Yield. An orange solid. Anal. Calcd. for $C_{39}H_{55}Cl_3FeMoNP_2$: C, 54.60; H, 6.46; N, 1.63. Found: C, 54.31; H, 6.33; N, 1.79.

[MoCl$_3$(2e)] (3e).

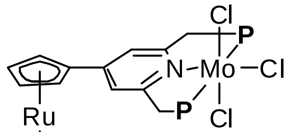

3e, P = P^{t}Bu$_2$

3e: 96% Yield. An orange solid. Anal. Calcd. for $C_{33}H_{51}Cl_3MoNP_2Ru$: C, 47.92; H, 6.22; N, 1.69. Found: C, 47.80; H, 6.41; N, 1.68.

Preparation of $[Mo(N_2)_2(2)]_2(\mu-N_2)$ (1).

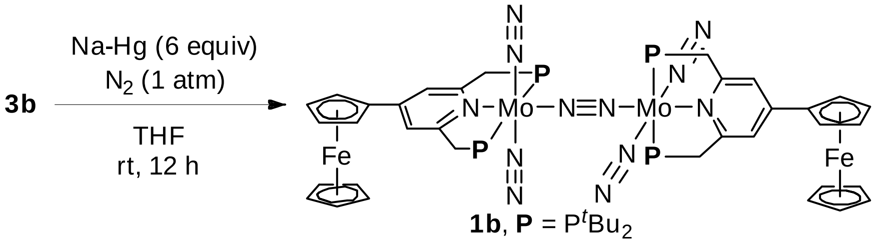

A typical procedure for the preparation of **1b**·$0.5C_6H_{14}\cdot C_6H_6$ is described below. To Na-Hg (0.5 wt%, 8.01 g, 1.74 mmol) were added THF (30 mL) and **3b** (232 mg. 0.297 mmol), and the mixture was stirred at room temperature for 12 h under N_2 (1 atm). The supernatant solution was decanted off and the residue was extracted with THF (10 mL). The combined extracts were dried *in vacuo*. After the residue was dissolved in benzene (5 mL), the solution was filtered through Celite and the filter cake was washed with benzene three times. After the combined filtrate was concentrated to ca. 5 mL, slow addition of hexane (20 mL) afforded a brown crystalline solid, which was collected by filtration and washed with hexane (1.5 mL) and dried *in vacuo* to afford **1b**·$0.5C_6H_{14}\cdot C_6H_6$ as a brown crystalline solid (178 mg, 0.110 mmol, 74% yield). The presence of 0.5 equiv of C_6H_{14} and 1 equiv of C_6H_6 was determined by ^{1}H NMR. ^{1}H NMR (C_6D_6) δ 7.00 (s, Py-*H*, 4H), 4.61 (t, *J* = 1.8 Hz, Cp-*H*, 4H), 4.23 (t, *J* = 1.8 Hz, Cp-*H*, 4H), 3.97 (s, Cp-*H*, 10H), 3.27 (br s, $CH_2P^tBu_2$, 8H), 1.45 (pseudo t, J_{P-H} = 5.8 Hz, $CH_2P^tBu_2$, 72H). $^{31}P\{^1H\}$ NMR (C_6D_6) δ 91.4 (s). IR (KBr, cm^{-1}) 1940 (s, ν_{NN}). IR (THF, cm^{-1}) 1944 (s, ν_{NN}). Raman (THF, cm^{-1}) 1888 (ν_{NN}). Anal. Calcd. for $C_{75}H_{115}Fe_2Mo_2N_{12}P_4$ (**1b**·$0.5C_6H_{14}\cdot C_6H_6$): C, 55.87; H, 7.19; N, 10.42. Found: C, 56.25; H, 7.54; N, 10.02. The slightly low content of nitrogen is considered to be due to the labile property of the coordinated molecular dinitrogen in **1**. No satisfactory of the elemental analysis is due to amounts of the solvent molecules, which depended on the isolation procedure of the complex. $^{13}C\{^1H\}$ NMR of the dinitrogen complexes **1** could not be measured clearly because of the low solubitlity of **1** in solvents.

[Mo(N$_2$)$_2$(2c)]$_2$(μ-N$_2$)·0.5C$_6$H$_{14}$ (1c·0.5C$_6$H$_{14}$).

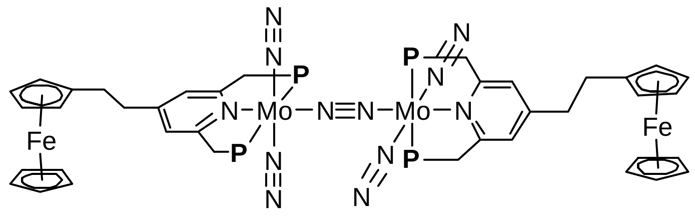

1c, **P** = P^{t}Bu$_2$

1c·0.5C$_6$H$_{14}$: 62% Yield. A green solid. The presence of 0.5 equiv of C$_6$H$_{14}$ was determined by ^{1}H NMR. ^{1}H NMR (C$_6$D$_6$): δ 6.55 (s, Py-*H*, 4H), 4.05 (s, Cp-*H*, 10H), 3.97 (t, *J* = 1.8 Hz, Cp-*H*, 4H), 3.93 (t, *J* = 1.8 Hz, Cp-*H*, 4H), 3.23 (br s, C*H$_2$*P^{t}Bu$_2$, 8H), 2.48 (br s, C*H$_2$*, 8H), 1.45 (pseudo t, *J* = 5.7 Hz, CH$_2$P^t*Bu$_2$*, 72H). ^{31}P{^{1}H} NMR (C$_6$D$_6$) δ 92.5 (s). IR (KBr, cm^{-1}) 1932 (s, ν$_{NN}$). IR (THF, cm^{-1}) 1939 (s, ν$_{NN}$). Raman (THF, cm^{-1}) 1894 (ν$_{NN}$). Anal. Calcd. for C$_{73}$H$_{117}$Fe$_2$Mo$_2$N$_{12}$P$_4$ (**1c**·0.5C$_6$H$_{14}$): C, 55.13; H, 7.42; N, 10.57. Found: C, 55.56; H, 7.28; N, 10.03.

[Mo(N$_2$)$_2$(2d)]$_2$(μ-N$_2$) (1d).

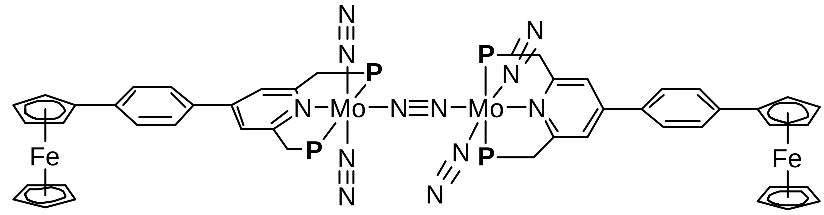

1d, **P** = P^{t}Bu$_2$

Recrystallization from toluene–hexane afforded **1d** as a brown solid. 32% Yield. ^{1}H NMR (THF-*d$_8$*) δ 7.62-7.48 (m, Ar-*H*, 12H), 4.75 (s, Cp-*H*, 4H), 4.33 (s, Cp-*H*, 4H), 4.01 (s, Cp-*H*, 10H), 3.45 (br s, C*H$_2$*P^{t}Bu$_2$, 8H), 1.32 (br s, CH$_2$P^t*Bu$_2$*, 72H). ^{31}P{^{1}H} NMR (THF-*d$_8$*) δ 92.7 (s). IR (KBr, cm^{-1}) 1947 (s, ν$_{NN}$). IR (THF, cm^{-1}) 1951 (s, ν$_{NN}$). Raman (THF, cm^{-1}) 1876 (ν$_{NN}$). Anal. Calcd. for C$_{78}$H$_{110}$Fe$_2$Mo$_2$N$_{12}$P$_4$: C, 57.01; H, 6.75; N, 10.23. Found: C, 57.63; H, 6.46; N, 7.30.

$[Mo(N_2)_2(2e)]_2(\mu\text{-}N_2)\cdot C_6H_{14}$ (1e·C_6H_{14}).

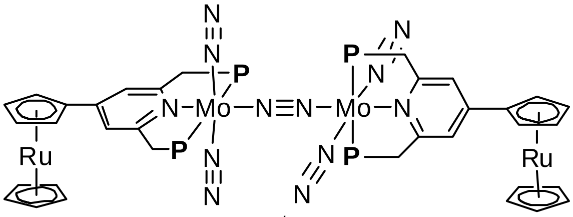

1e, **P** = P^tBu_2

1e·C_6H_{14}: 52% Yield. A brown solid. ^{1}H NMR (THF-d_8) δ 7.15 (s, Py-*H*, 4H), 5.13 (t, *J* = 1.6 Hz, Cp-*H*, 4H), 4.67 (t, *J* = 1.6 Hz, Cp-*H*, 4H), 4.40 (s, Cp-*H*, 10H), 3.34 (br s, C$H_2P^tBu_2$, 8H), 1.29 (pseudo t, *J* = 5.7 Hz,$CH_2P^tBu_2$, 72H). $^{31}P\{^1H\}$ NMR (THF-d_8) δ 93.1 (s). IR (KBr, cm^{-1}) 1936 (s, ν_{NN}). IR (THF, cm^{-1}) 1944 (s, ν_{NN}). Raman (THF, cm^{-1}) 1888 (ν_{NN}). Anal. Calcd. for $C_{72}H_{116}Mo_2N_{12}P_4Ru_2$ (**1e**·C_6H_{14}): C, 51.85; H, 7.01; N, 10.08. Found: C, 52.24; H, 6.48; N, 9.29.

X-ray Crystallography.

Single crystals of **1b** suitable for X-ray crystallography were obtained as **1b**·C_6H_{14} by recrystallization from benzene–hexane. Crystallographic data of **1b**·C_6H_{14} (CCDC 1048952), **1e**·C_6H_{14} (CCDC 1048953), **3b**·C_6H_{14} (CCDC 1048954), **3c**·CH_2Cl_2·$0.5C_6H_{14}$ (CCDC 1048955), and **3d**·CH_2Cl_2 (CCDC 1048956) are summarized in Table S6–7. Selected bond lengths and angles are summarized in Tables S1–S5, and their ORTEP drawings are shown in Figures S1–S5. Diffraction data for **1b**·C_6H_{14}, **1e**·C_6H_{14}, **3b**·C_6H_{14}, **3c**·CH_2Cl_2·$0.5C_6H_{14}$, and **3d**·CH_2Cl_2 were collected for the 2θ range of 5° to 55° at −75 °C (for **1e**·C_6H_{14}, **3c**·CH_2Cl_2·$0.5C_6H_{14}$, and **3d**·CH_2Cl_2), −80 °C (for **1b**·C_6H_{14}) or −100 °C (for **3b**·C_6H_{14}) on a Rigaku RAXIS RAPID imaging plate area detector with graphite-monochromated Mo Kα radiation (λ = 0.71075 Å), with VariMax optics. Intensity data were collected for Lorenz-polarization effects and for empirical absorption (REQAB). The structure solution and refinements were carried out by using the *CrystalStructure* crystallographic software package.[S11] The positions of the non-hydrogen atoms were determined by direct methods (SIR 97[S12] for **1b**·C_6H_{14}, **1e**·C_6H_{14} , **3c**·CH_2Cl_2·$0.5C_6H_{14}$, and **3d**·CH_2Cl_2, SIR 2002[S13] for **3b**·C_6H_{14}) and subsequent Fourier syntheses (DIRDIF-99[S14]) and were refined F_o^2 using all unique reflections by full-matrix least-squares with anisotropic thermal parameters except for a few solvated molecules (C67–C73 for **1b**·C_6H_{14}; C67–C72 for **1e**·C_6H_{14}; C34–C39 for **3b**·C_6H_{14}; Cl7-Cl10, C71-C78 for **3c**·CH_2Cl_2·$0.5C_6H_{14}$), which were refined isotropically. All the other hydrogen atoms were placed at the calculated positions with fixed isotropic parameter.

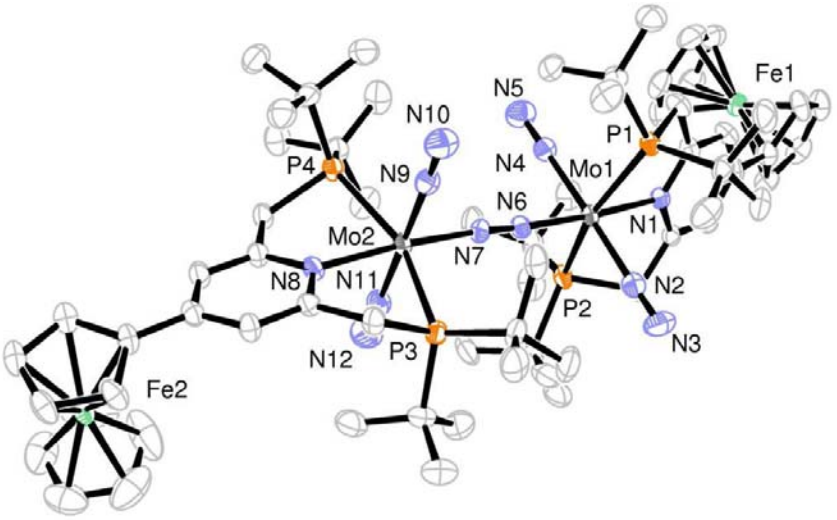

Figure S1. ORTEP drawing of **1b**·C_6H_{14}. Hydrogen atoms and solvated molecules are omitted for clarity.

Table S1. Selected Bond Lengths (Å) and Angles (deg) for **1b**·C_6H_{14}.

Mo(1)–N(1)	2.182(3)	Mo(1)–N(2)	2.013(3)
Mo(1)–N(4)	2.026(3)	Mo(1)–N(6)	2.030(3)
N(2)–N(3)	1.131(4)	N(4)–N(5)	1.111(4)
N(6)–N(7)	1.146(4)	Mo(1)–P(1)	2.4470(10)
Mo(1)–P(2)	2.5035(9)	Mo(2)–N(7)	2.015(3)
Mo(2)–N(8)	2.180(3)	Mo(2)–N(9)	2.004(3)
Mo(2)–N(11)	2.024(3)	N(9)–N(10)	1.124(4)
N(11)–N(12)	1.114(4)	Mo(2)–P(3)	2.4694(9)
Mo(2)–P(4)	2.4733(9)		
Mo(1)–N(2)–N(3)	178.3(3)	Mo(1)–N(4)–N(5)	179.3(3)
Mo(1)–N(6)–N(7)	176.9(3)	Mo(2)–N(7)–N(6)	173.8(3)
Mo(2)–N(9)–N(10)	177.5(3)	Mo(2)–N(11)–N(12)	179.6(3)
P(1)–Mo(1)–P(2)	156.91(3)	P(3)–Mo(2)–P(4)	157.52(3)

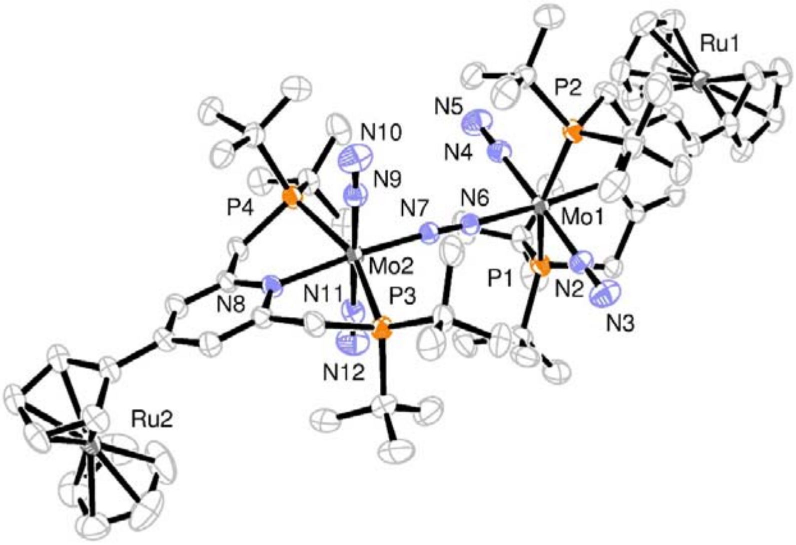

Figure S2. ORTEP drawing of **1e**·C_6H_{14}. Hydrogen atoms and solvated molecules are omitted for clarity.

Table S2. Selected Bond Lengths (Å) and Angles (deg) for **1e**·C_6H_{14}.

Mo(1)–N(1)	2.166(3)	Mo(1)–N(2)	2.005(3)
Mo(1)–N(4)	2.022(3)	Mo(1)–N(6)	2.027(3)
N(2)–N(3)	1.119(5)	N(4)–N(5)	1.118(5)
N(6)–N(7)	1.139(4)	Mo(1)–P(1)	2.4995(8)
Mo(1)–P(2)	2.4493(9)	Mo(2)–N(7)	2.010(3)
Mo(2)–N(8)	2.172(3)	Mo(2)–N(9)	2.003(3)
Mo(2)–N(11)	2.026(3)	N(9)–N(10)	1.128(4)
N(11)–N(12)	1.121(4)	Mo(2)–P(3)	2.4656(10)
Mo(2)–P(4)	2.4726(10)		
Mo(1)–N(2)–N(3)	178.8(3)	Mo(1)–N(4)–N(5)	179.4(3)
Mo(1)–N(6)–N(7)	177.3(3)	Mo(2)–N(7)–N(6)	174.3(3)
Mo(2)–N(9)–N(10)	177.2(3)	Mo(2)–N(11)–N(12)	179.7(3)
P(1)–Mo(1)–P(2)	157.05(4)	P(3)–Mo(2)–P(4)	157.53(4)

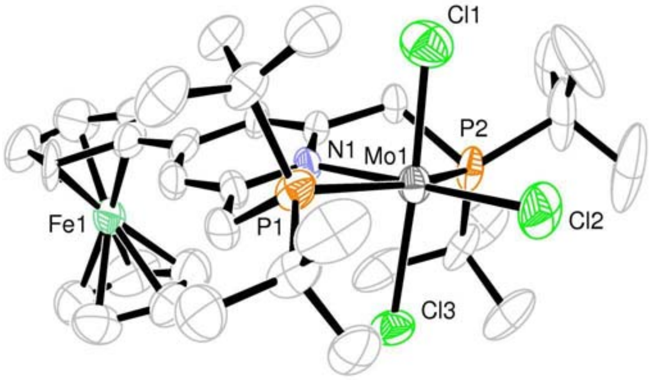

Figure S3. ORTEP drawing of **3b**·C_6H_{14}. Hydrogen atoms and solvated molecules are omitted for clarity.

Table S3. Selected Bond Lengths (Å) and Angles (deg) for **3b**·C_6H_{14}.

Mo(1)–Cl(1)	2.315(4)	Mo(1)–P(1)	2.582(3)
Mo(1)–Cl(2)	2.419(2)	Mo(1)–P(2)	2.603(3)
Mo(1)–Cl(3)	2.439(3)	Mo(1)–N(1)	2.189(4)
P(1)–Mo(1)–P(2)	155.51(7)		

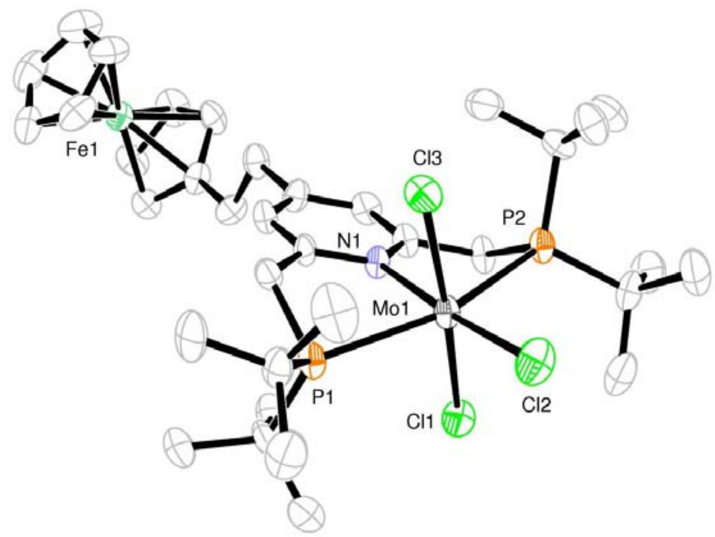

Figure S4. **3c**·CH_2Cl_2·0.5C_6H_{14}. One of the two crystallographically independent molecules. Hydrogen atoms and solvated molecules are omitted for clarity.

Table S4. Selected Bond Lengths (Å) and Angles (deg) for **3c**·CH_2Cl_2·0.5C_6H_{14}.

Mo(1)–Cl(1)	2.396(2)	Mo(1)–P(1)	2.595(3)
Mo(1)–Cl(2)	2.4043(19)	Mo(1)–P(2)	2.590(3)
Mo(1)–Cl(3)	2.432(2)	Mo(1)–N(1)	2.171(5)
Mo(2)–Cl(4)	2.421(3)	Mo(2)–P(3)	2.596(2)
Mo(2)–Cl(5)	2.3880(18)	Mo(2)–P(4)	2.604(2)
Mo(2)–Cl(6)	2.407(3)	Mo(2)–N(2)	2.187(5)
P(1)–Mo(1)–P(2)	157.25(6)	P(3)–Mo(2)–P(4)	156.46(6)

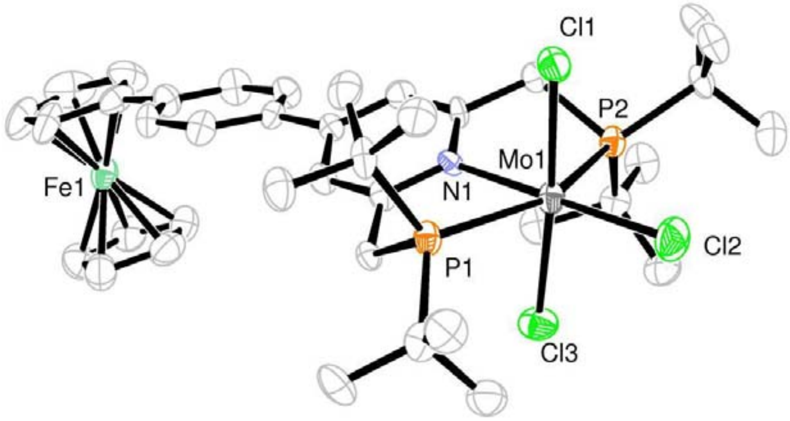

Figure S5. ORTEP drawing of **3d**·CH_2Cl_2. Hydrogen atoms and solvated molecules are omitted for clarity.

Table S5. Selected Bond Lengths (Å) and Angles (deg) for **3d**·CH_2Cl_2.

Mo(1)–Cl(1)	2.421(3)	Mo(1)–P(1)	2.6593(19)
Mo(1)–Cl(2)	2.3989(16)	Mo(1)–P(2)	2.6019(19)
Mo(1)–Cl(3)	2.421(3)	Mo(1)–N(1)	2.207(4)
P(1)–Mo(1)–P(2)	155.36(6)		

Table S6. X-ray Crystallographic Data for **1b**·C_6H_{14}, **1e**·C_6H_{14}, and **3b**·C_6H_{14}.

	**1b**·C_6H_{14}	**1e**·C_6H_{14}	**3b**·C_6H_{14}
CCDC	1048952	1048953	1048954
chemical formula	$C_{72}H_{116}Fe_2Mo_2N_{12}P_4$	$C_{72}H_{116}Mo_2N_{12}P_4Ru_2$	$C_{39}H_{65}Cl_3FeMoNP_2$
formula weight	1577.26	1667.70	868.04
dimensions of crystals	0.20 × 0.15 × 0.15	0.15 × 0.15 × 0.03	0.20 × 0.10 × 0.10
crystal system	triclinic	triclinic	monoclinic
space group	$P\overline{1}$	$P\overline{1}$	Cc
a, Å	14.749(3)	14.7212(3)	21.922(1)
b, Å	15.266(3)	15.3604(3)	15.8520(7)
c, Å	18.081(4)	18.0121(4)	14.0070(6)
α, deg	72.467(5)	72.7582(7)	90
β, deg	83.794(4)	84.4816(7)	121.6313(9)
γ, deg	81.014(4)	81.1096(7)	90
V, Å^3	3826(2)	3837.7(2)	4144.4(3)
Z	2	2	4
ρ_{calcd}, g cm^{-3}	1.369	1.443	1.391
$F(000)$	1656.00	1728	1822.00
μ, cm^{-1}	8.227	8.363	9.497
trans. factors range	0.712-0.884	0.704-0.975	0.609-0.909
no. reflections measured	36283	38040	19915
no. unique reflections	17216(R_{int} = 0.0495)	17440 (R_{int} = 0.0348)	8724 (R_{int} = 0.0524)
no. parameters refined	905	901	446
$R1$ ($I > 2\sigma(I)$)[a]	0.0452	0.0424	0.0650
$wR2$ (all data)[b]	0.0934	0.0746	0.1225
GOF (all data)[c]	1.013	1.007	1.038
Flack parameters			0.08(3)
max diff peak / hole, e Å^{-3}	1.28/-0.74	1.27/-0.96	1.52/-2.22

[a] $R1 = \Sigma||F_o|-|F_c||/\Sigma|F_o|$. [b] $wR2 = [\Sigma w(F_o^2-F_c^2)^2/\Sigma w(F_o^2)^2]^{1/2}$, $w = 4F_o^2/q\sigma(F_o^2)$ [q = 2.5 (**1b**·C_6H_{14}); q = 3.7(**1e**·C_6H_{14}); q = 3.9 (**3b**·C_6H_{14})]. [c] GOF = $[\Sigma w(F_o^2-F_c^2)^2/(N_o-N_{params})]^{1/2}$.

Table S7. X-ray Crystallographic Data for **3c**·CH_2Cl_2·0.5C_6H_{14}, and **3d**·CH_2Cl_2.

	**3c**·CH_2Cl_2·0.5C_6H_{14}	**3d**·CH_2Cl_2
CCDC	1048955	1048956
chemical formula	$C_{78}H_{128}Cl_{10}Fe_2Mo_2N_2P_4$	$C_{40}H_{57}Cl_5FeMoNP_2$
formula weight	1875.88	942.90
dimensions of crystals	0.30 × 0.10 × 0.05	0.20× 0.10 × 0.10
crystal system	triclinic	monoclinic
space group	$P\overline{1}$	*Cc*
a, Å	13.2456(6)	18.938(2)
b, Å	15.6143(6)	19.521(2)
c, Å	24.227(2)	13.7984(8)
α, deg	90.538(2)	90
β, deg	102.695(2)	120.860(1)
γ, deg	112.964(1)	90
V, Å^3	4475.5(4)	4379.0(5)
Z	2	4
ρ_{calcd}, g cm^{-3}	1.392	1.430
F(000)	1952.00	1948.00
μ, cm^{-1}	10.004	10.231
trans. factors range	0.549-0.951	0.585-0.903
no. reflections measured	44300	19348
no. unique reflections	20334 (R_{int} = 0.0886)	8553 (R_{int} = 0.0546)
no. parameters refined	951	509
$R1$ ($I > 2\ \sigma(I)$)^a	0.0927	0.0503
$wR2$ (all data)^b	0.1751	0.0968
GOF (all data)^c	0.981	1.012
Flack parameters		-0.01(3)
max diff peak / hole, e Å^{-3}	4.17/-3.59	3.74/-4.20

^{a} $R1 = \Sigma||F_o|-|F_c||/\Sigma|F_o|$. ^{b} $wR2 = [\Sigma w(F_o^2-F_c^2)^2/\Sigma w(F_o^2)^2]^{1/2}$, $w = 4F_o^2/\ q\sigma(F_o^2)$ [q = 3.8 (**3c**·CH_2Cl_2·0.5C_6H_{14}); q = 3.3 (**3d**·CH_2Cl_2)]. ^{c} GOF = $[\Sigma w(F_o^2-F_c^2)^2/(N_o-N_{params})]^{1/2}$.

Table S8. Selected Bond Distances of Molybdenum Trichloride Complexes.

	**3a** (R = H)	**3b** (R = Fc)	**3c** (R = Et^{Fc})	**3d** (R = Ph^{Fc})
Distances (Å, mean)				
Mo–Cl	2.41	2.39	2.41	2.41
Mo–N	2.20	2.19	2.18	2.21
Mo–P	2.61	2.59	2.60	2.63

Table S9. Selected Bond Distances and Angles of Molybdenum Dinitrogen Complexes.

	**1a** (R = H)	**1b** (R = Fc)	**1e** (R = Rc)
Distances (Å, mean)			
N–N (bridging)	1.15	1.15	1.14
N–N (terminal)	1.12	1.12	1.12
Mo–N (bridging)	2.02	2.02	2.02
Mo–N (terminal)	2.03	2.02	2.01
Mo–N (pyridine)	2.18	2.18	2.17
Mo–P	2.47	2.47	2.47
Angles (deg, mean)			
Mo–N–N (bridging)	178	175	176
Mo–N–N (terminal)	179	179	179
P–Mo–P	157	157	157

Electrochemical Study

Cyclic voltammograms were recorded on an ALS/Chi model 610C electrochemical analyzer with platinum working electrode in THF containing 1 mM of sample and 0.1 M of $[NBu_4]BAr^F_4$ as a supporting electrolyte at a scan rate of 0.1 V/s at room temperature. All potentials were measured against an $Ag^{0/+}$ electrode and converted to the values vs $FeCp_2^{0/+}$.

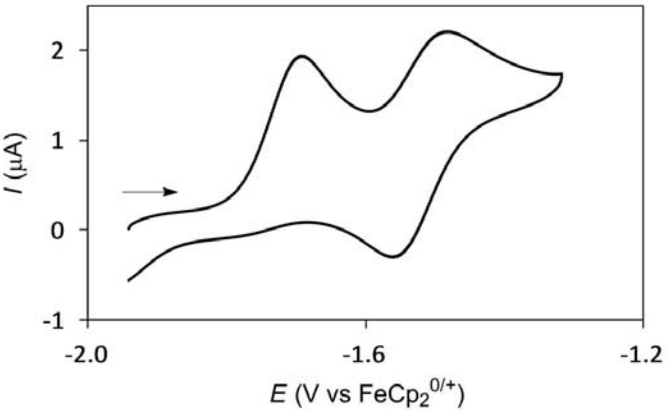

Figure S6. Cyclic voltammogram of dinitrogen complex **1b**. In cyclic voltammogram of **1b**, anodic scan showed two successive oxidation waves (E^1_{pa} = −1.69 V and E^2_{pa} = −1.48 V) and the reverse cathodic scan showed one reduction wave (E_{pc} = −1.56 V).

Table S10. Cyclic voltammetry data for dimolybdenum dinitrogen complexes **1a**–**1e**

compound	R	E^1_{pa} (V)	E^2_{pa} (V)	E_{pc} (V)
**1a**	H	−1.67	−1.47	−1.55
**1b**	Fc	−1.69	−1.48	−1.56
**1c**	Et^{Fc}	−1.69	−1.48	−1.57
**1d**	Ph^{Fc}	−1.64	−1.44	−1.52
**1e**	Rc	−1.69	−1.49	−1.57

Spectroscopic Study

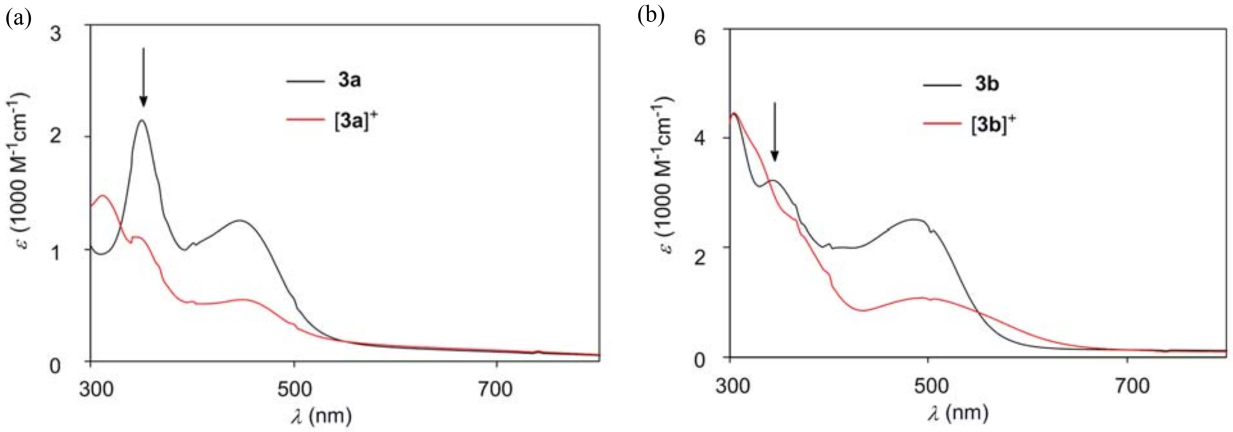

Figure S7. UV-Vis spectra of (a) **3a** and [**3a**]$^+$, (b) **3b** and [**3b**]$^+$. The spectra were recorded on a JASCO V-630 spectrophotometer. Oxidative spectroelectrochemistry was perfomed on BAS 100B/W electrochemical analyzer in a thin-layer cell (optical length: 0.1 cm) with platinum working electrode in THF containing 1 mM of sample and 0.1 M of $[NBu_4]BAr^F{}_4$ as a supporting electrolyte. The spectra of monocation species [**3a**]$^+$ and [**3b**]$^+$ were obtained at +0.29 V vs $FeCp_2{}^{0/+}$.

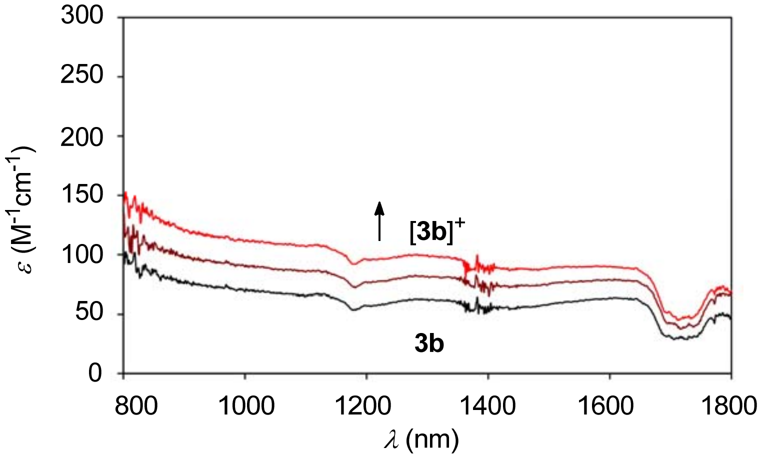

Figure S8. Near-IR spectra of **3b** (black), partially oxidized **3b** (brown) and [**3b**]$^+$ (red). The spectra were recorded on a Simadzu-3100PC spectrophotometer. Oxidative spectroelectrochemistry was perfomed on BAS 100B/W electrochemical analyzer in a thin-layer cell (optical length: 0.1 cm) with platinum working electrode in THF containing 4 mM of sample and 0.1 M of $[NBu_4]BAr^F{}_4$ as a supporting electrolyte. The spectra of oxidized species were obtained at +0.29 V vs $FeCp_2{}^{0/+}$.

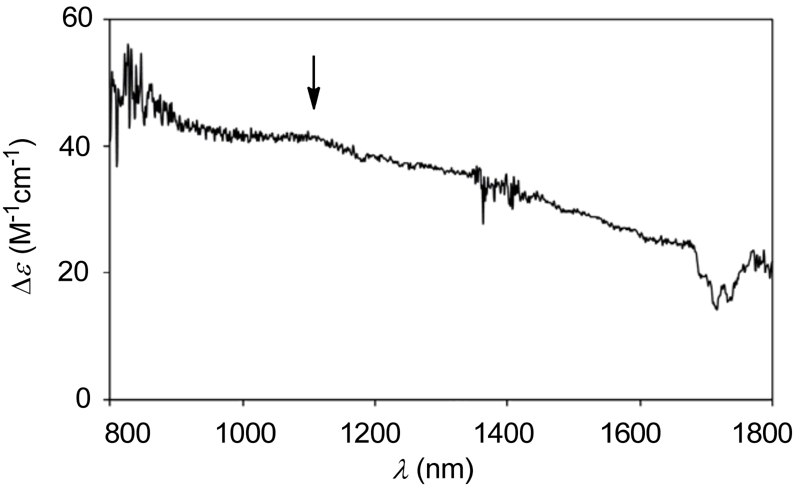

Figure S9. Difference between the near-IR spectrum of [**3b**]$^+$ and that of **3b**.

The electronic coupling parameter H_{ab} can be calculated using an equation[S15] defined as follows:

$H_{ab} = 0.0206 \times (\nu_{max}\varepsilon_{max}\Delta\nu_{1/2})^{1/2}/r_{ab}$

The parameters for this equation can be obtained from Figure S9 and the crystal structure of complex **3b**.

The distance between the molybdenum atom and the iron atom: $r_{ab} = 7.70$ Å

The wave length of the maximum absorbance: $\lambda_{max} = 1100$ nm

The wave number of the maximum absorbance: $\nu_{max} = 9.09 \times 10^3$ cm^{-1}

The maximum absorbance: $\varepsilon_{max} = 4.2\times 10$ M^{-1}cm^{-1}

The full width at half maximum of the band: $\nu_{1/2} = 3.5 \times 10^3$ cm^{-1}

Using these parameters, $H_{ab} = 9.8 \times 10$ cm^{-1}

Catalytic reduction of dinitrogen to ammonia under N_2 atmosphere (1 atm).

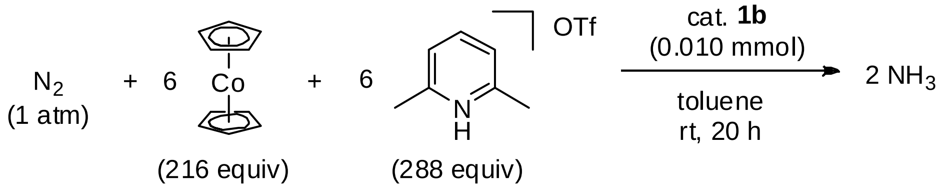

A typical experimental procedure for the catalytic reduction of dinitrogen into ammonia using **1b** is described below.

In a nitrogen-filled glove box, to a mixture of **1b**·$0.5C_6H_{14}$·C_6H_6 (15.9 mg, 0.010 mmol) and [LutH]OTf (740.7 mg, 2.88 mmol) in a 50 mL Schlenk flask was added toluene (1.0 mL). Then a solution of $CoCp_2$ (408.4 mg, 2.16 mmol) in toluene (4.0 mL) was slowly added to the stirred mixture in the Schlenk flask with a syringe pump at a rate of 4.0 mL per hour. After the addition of $CoCp_2$, the mixture was further stirred at room temperature for 19 h. The amount of dihydrogen of the catalytic reaction was determined by GC analysis. The reaction mixture was evaporated under reduced pressure, and the distillate was trapped in dilute H_2SO_4 solution (0.5 M, 10 mL). Potassium hydroxide aqueous solution (30 wt%; 5 mL) was added to the residue, and the mixture was distilled into another dilute H_2SO_4 solution (0.5 M, 10 mL). NH_3 present in each of the H_2SO_4 solutions was determined by the indophenol method.[S16] The amount of ammonia was 0.054 mmol of NH_3 collected before base distillation of the reaction mixture and 0.333 mmol of NH_3 collected after base distillation to fully liberate NH_3, respectively. The total amount of ammonia was 0.387 mmol (38.7 equiv per **1b**).

Table S11. Catalytic reduction of molecular dinitrogen with [LutH]OTf and a reductant in the presence of **1b**.[a]

$$\underset{(1\ \text{atm})}{N_2} + \underset{(216\ \text{equiv})}{6\ \text{reductant}} + \underset{(288\ \text{equiv})}{6\ [\text{LutH}]\text{OTf}} \xrightarrow[\text{toluene, rt, 20 h}]{\text{cat. } \mathbf{1b}} 2\ NH_3$$

run	reductant	$E_{1/2}$[b]	NH_3 (equiv)[c]	NH_3 (%)[d]	H_2 (equiv)[c]	H_2 (%)[d]
1	$CoCp_2$	−1.33	39	54	35	32
2	$CrCp*_2$	−1.55	24	33	43	40

To a suspension of **1b** (0.010 mmol) and [LutH]OTf (288 equiv to the catalyst) in toluene (1.0 mL) was added a solution of reductant (216 equiv to the catalyst) at room temperature over a period of 1 h, followed by stirring at room temperature for another 19 h under an atomospheric pressure of dinitrogen. [b] V vs $FeCp_2^{0/+}$. Electrochemical data ($E_{1/2}$) of the reductant was measured in THF, with $[NBu_4]BAr^F_4$ as a supporting electrolyte. [c] Mol equiv to the catalyst. [d] Yield based on $CoCp_2$.

Table S12. Catalytic reduction of molecular dinitrogen with $CoCp_2$ and a proton source in the presence of **1b**.[a]

$$\underset{(1\ \text{atm})}{N_2} + \underset{(216\ \text{equiv})}{6\ CoCp_2} + \underset{(288\ \text{equiv})}{6\ \text{proton source}} \xrightarrow[\text{toluene, rt, 20 h}]{\text{cat. } \mathbf{1b}} 2\ NH_3$$

run	proton source	pK_a[b]	NH_3 (equiv)[c]	NH_3 (%)[d]	H_2 (equiv)[c]	H_2 (%)[d]
1	[LutH]OTf	6.77	39	54	35	32
2	[PicH]OTf	5.97	7	9	58	53
3	[ColH]OTf	7.48	10	14	53	49

[a] To a suspension of **1b** (0.010 mmol) and a proton source (288 equiv to the catalyst) in toluene (1.0 mL) was added a solution of $CoCp_2$ (216 equiv to the catalyst) at room temperature over a period of 1 h, followed by stirring at room temperature for another 19 h under an atomospheric pressure of dinitrogen. [b] pK_a value of proton source in H_2O (reference S17). [c] Mol equiv to the catalyst. [d] Yield based on $CoCp_2$.

Catalytic reduction of dinitrogen to ammonia using larger amounts of reductant and proton source.

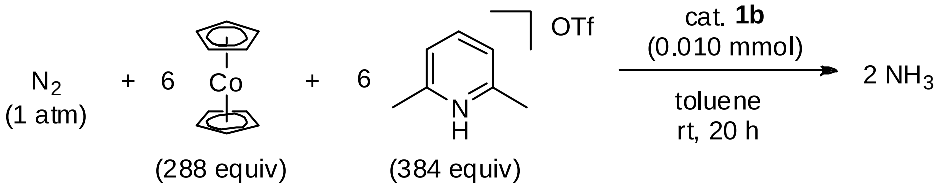

In a nitrogen-filled glove box, to a mixture of **1b**·$0.5C_6H_{14}$·C_6H_6 (15.8 mg, 0.010 mmol) and [LutH]OTf (493.8 mg, 1.92 mmol) in a 50 mL Schlenk flask was added toluene (1.0 mL). Then a solution of $CoCp_2$ (544.7 mg, 2.88 mmol) in toluene (6.0 mL) was slowly added to the stirred mixture in the Schlenk flask with a syringe pump at a rate of 6.0 mL per hour. After 0.5 h, stirring was stopped. [LutH]OTf (493.8 mg, 1.92 mmol) was added to the Schlenk flask in one portion. The remaining solution of $CoCp_2$ was added again to the reaction mixture with a syringe pump at a rate of 6.0 mL per hour. After addition of $CoCp_2$, the mixture was further stirred at room temperature for 19 h. The total amount of ammonia was 0.445 mmol (45 equiv per **1b**).

Catalytic Reduction of Dinitrogen to Ammonia under $^{15}N_2$.

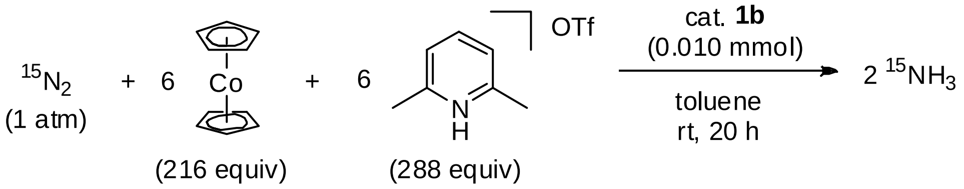

In a 50 mL Schlenk were placed **1b**·$0.5C_6H_{14}$·C_6H_6 (16.0 mg, 0.010 mmol) and [LutH]OTf (740.7 mg, 2.88 mmol). The Schlenk flask was evacuated and then filled with $^{15}N_2$. Toluene (1.0 mL) was added to the Schlenk flask. Then a solution of $CoCp_2$ (408.5 mg, 2.16 mmol) in toluene (4.0 mL) was added to the stirred mixture in the Schlenk flask with a syringe pump at a rate of 4.0 mL per hour. After the addition of $CoCp_2$, the mixture was further stirred at room temperature for 19 h. The total amount of ammonia was 0.204 mmol (20.4 equiv per **1b**.)

In separate run, an NMR sample of the ammonium salt was prepared as follows. In a 50 mL Schlenk were placed **1b**·$0.5C_6H_{14}$·C_6H_6 (16.1 mg, 0.010 mmol) and [LutH]OTf (741.0 mg, 2.88 mmol). The Schlenk flask was evacuated and then filled with $^{15}N_2$. Toluene (1.0 mL) was added to the Schlenk flask. A solution of $CoCp_2$ (408.4 mg, 2.16 mmol) in toluene (4.0 mL) was then added to the mixture in the Schlenk flask with a syringe pump at a rate of 4.0 mL per hour. After the addition of $CoCp_2$, the mixture was further stirred at room temperature for 19 h. After catalytic reduction was completed, the volatiles were collected by trap-to-trap distillation. To the residual solid was added a solution of KO^{t}Bu (4 mmol) in THF−MeOH (4 mL/2 mL) and the mixture was stirred at room temperature for 30 min. The volatile components in the mixture were collected to the Schlenk flask to which was added HCl in Et_2O (2 M, 5 mL). The solvent was dried in *vacuo* to afford a solid which contains $^{15}NH_4Cl$ and [LutH]Cl. ^{1}H NMR (DMSO-d_6) δ 7.55 (d, $J_{N\text{-}H}$ = 71.0 Hz, $^{15}NH_4Cl$). $^{15}N\{^1H\}$ NMR (DMSO-d_6) δ −351.2 (s, $^{15}NH_4Cl$).

Time profiles of the formation of ammonia.

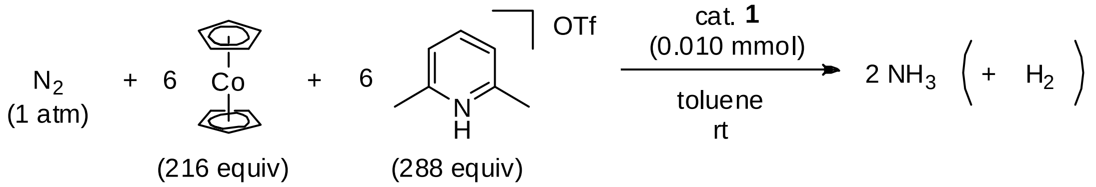

A typical procedure is as follows. In a 50 mL Schlenk flask were placed the catalyst (0.010 mmol) and [LutH]OTf (2.88 mmol). Toluene (1.0 mL) was added under N_2 (1 atm), and then a solution of $CoCp_2$ (2.16 mmol) in toluene (4.0 mL) was slowly added to the stirred mixture in the Schlenk flask with a syringe pump at a rate of 4.0 mL per hour. After the indicated time, the amount of molecular dihydrogen in the catalytic reaction was determined by GC analysis. Then potassium hydroxide aqueous solution (30 wt%; 5 mL) was added to the mixture, and the mixture was distilled into dilute H_2SO_4 solution (0.5 M, 10 mL). The amount of ammonia present in the H_2SO_4 solutions was determined by the indophenol method.[S16] The results are summarized in Table S13..

Table S13. The amounts of ammonia and molecular dihydrogen catalyzed by **1**.[a]

	**1a** (R = H)		**1b** (R = Fc)		**1h** (R = MeO)	
time (h)	NH_3 (equiv)	H_2 (equiv)	NH_3 (equiv)	H_2 (equiv)	NH_3 (equiv)	H_2 (equiv)
0.33	7	3	6	3		
0.67	15	14	16	7		
1	17	23	23	17		
2	25	38	26	27	14	10
4			31	30	22	21
6			33	30		
8					30	35
20	23	46	37	36	34	36

[a] Based on catalyst **1**.

Computational Methods.

All DFT calculations were performed by using the Gaussian 09 program.[S18] Similar to our previous studies,[S5,S19] geometry optimizations were carried out with the B3LYP* functional. The B3LYP* functional is a reparametrized version of the B3LYP hybrid functional[S20] developed by Reiher and co-workers for proper estimation of the energy difference between different spin states.[S21] The LANL2DZ[S22] and 6-31G(d)[S23] basis sets were employed for metal atoms and the other atoms, respectively. For the evaluation of the intramolecular electron transfer in $[MoCl_3(4\text{-}Fc\text{-}PNP)]^+$ **$[3b]^+$** and $[Mo(PNP)(OTf)(NNH_3)–NN–Mo(PNP)(N_2)_2]^+$ **IIb**, time-dependent BP86 (TD-BP86) calculations[S24] were performed to estimate 50 electronic transitions from the lowest-energy transition. In the TD-BP86 calculations, the SDD (Stuttgart/Dresden pseudopotentials) basis set[S25] was used instead of the LANL2DZ basis set. For the evaluation of electron transitions with the TD-BP86 method, we previously demonstrated that the reproducibility of the UV-vis spectrum of a dimolybdenum dinitrogen complex $[Cp^*Mo(depf)]_2(\mu\text{-}N_2)$ (Cp* = pentamethylcyclopentadienyl, depf = 1,1'-bis(diethylphosphino)ferrocene).[S26] Electron density difference maps (EDDMs) were produced based on the data of singly-excited configurations in the TD-BP86 results by using the GaussSum program.[S27] Cartesian coordinates of **$[3b]^+$**, **$[3b]^{2+}$**, and **IIb** are listed in Tables S14-S16.

Table S14. Cartesian coordinate of **[3b]**$^+$ in the triplet state. Units are presented in Å.

SCF energy = -3595.71609878 hartree

ZPE = 0.774358 hartree

Atom	X	Y	Z
Mo	2.249496089	0.593703738	0.172782708
Fe	-5.341338227	-1.639310961	-0.566027645
P	1.665650883	0.637710995	2.775405262
P	1.731750353	0.444751106	-2.440319725
N	0.030175589	0.35645148	0.154737885
Cl	1.786416945	2.915125443	0.046163579
Cl	2.271536118	-1.776468623	0.30199642
Cl	4.557667758	0.828616468	0.180265576
C	0.968401979	2.245648804	3.59876121
C	2.035849103	3.36096008	3.541706195
C	0.537535358	2.022158023	5.064620348
C	-0.291867977	2.702128888	2.828623733
C	2.784136135	-0.334011837	4.021952766
C	3.818212626	0.635841105	4.633121539
C	1.953581571	-1.017277158	5.130079022
C	3.568160657	-1.434113218	3.277698284
C	0.157440492	-0.444299689	2.507639553
C	-0.633289901	-0.085067583	1.26718677
C	-2.014264497	-0.228316613	1.273421062
C	-2.787546475	0.077043815	0.13668271
C	-2.075047302	0.529773357	-0.99098583
C	-0.693442916	0.659155073	-0.967410862

C	-4.240300719	-0.030720744	0.1431573
C	-5.059133327	-0.592665157	1.193781538
C	-6.422490498	-0.490461368	0.801445941
C	-6.472486016	0.112022391	-0.493175062
C	-5.140075785	0.383414524	-0.911158008
C	-4.08805104	-3.117909371	-1.32571436
C	-4.867446535	-3.638628999	-0.246774215
C	-6.247092855	-3.505702168	-0.59849316
C	-6.318670894	-2.900884045	-1.891068198
C	-4.983012975	-2.658919699	-2.341229976
C	0.026062331	1.183818708	-2.192423247
C	2.641396268	1.638749837	-3.662959629
C	1.711332411	2.12909212	-4.793849631
C	3.875011267	0.915251845	-4.244128996
C	3.151118252	2.881212301	-2.904204179
C	1.409355159	-1.267969899	-3.282578649
C	0.977583229	-1.133758164	-4.758793356
C	2.684315042	-2.136191786	-3.196851575
C	0.256825191	-1.985321624	-2.542754753
H	2.926008524	3.121658804	4.1306257
H	2.347810791	3.580647191	2.517001355
H	1.602228168	4.278651471	3.963573044
H	-0.197148623	1.214164102	5.170599468
H	1.37683638	1.823529779	5.736761307
H	0.054593549	2.944282697	5.417354792
H	-0.60273861	3.673404842	3.237716197
H	-0.118438569	2.839921605	1.759080939
H	-1.132167317	2.011839604	2.967738161

H	4.402350405	1.152011883	3.860751271
H	3.372422002	1.384688788	5.294006238
H	4.523124235	0.049862333	5.238361146
H	2.645187823	-1.566255127	5.784516809
H	1.397237265	-0.31888027	5.758692331
H	1.250594276	-1.754049899	4.721743971
H	2.918663907	-2.213512371	2.86935209
H	4.182041453	-1.034541245	2.46425345
H	4.240501725	-1.91316719	4.003202225
H	0.549816646	-1.464017854	2.390919816
H	-0.502974393	-0.443933309	3.382039332
H	-2.496207946	-0.567924617	2.185188291
H	-2.60515455	0.786625833	-1.903073228
H	-4.70587665	-1.031223264	2.11929046
H	-7.273589664	-0.844028904	1.371504994
H	-7.367660603	0.294776246	-1.075823628
H	-4.859877503	0.838134232	-1.85371115
H	-3.005897388	-3.051235382	-1.353538028
H	-4.4830594	-4.046371129	0.681024169
H	-7.092458881	-3.786229343	0.019321889
H	-7.227038867	-2.63915349	-2.421341905
H	-4.702012192	-2.193917436	-3.279242052
H	0.198648505	2.26075108	-2.059941357
H	-0.608665442	1.061637201	-3.077238881
H	2.277539825	2.833762705	-5.418806864
H	1.353531943	1.330903293	-5.447710972
H	0.843772458	2.67643283	-4.40469547
H	4.535913406	0.534067858	-3.455407361

H	3.615456503	0.090119748	-4.913289876
H	4.45356666	1.639494425	-4.833493771
H	2.339465755	3.501244305	-2.513125064
H	3.818515389	2.622794405	-2.076170069
H	3.720068887	3.49604797	-3.615751118
H	0.090609209	-0.500048517	-4.883794355
H	1.771828844	-0.756650786	-5.40893481
H	0.711887893	-2.135599097	-5.124688325
H	2.996714598	-2.30516581	-2.162846963
H	2.468142372	-3.115991	-3.645527143
H	3.526155169	-1.705510004	-3.746619612
H	0.162075077	-2.994815594	-2.966813345
H	0.439207683	-2.096386652	-1.471418422
H	-0.705263588	-1.480458334	-2.692134018

Table S15. Cartesian coordinate of **[3b]**$^{2+}$ in the quartet state. Units are presented in Å.

SCF energy = -3595.40123802 hartree

ZPE = 0.773413 hartree

Atom	Coordinates (Angstroms) X	Y	Z
Mo	2.302422205	0.615262879	0.204955896
Fe	-5.542576049	-1.687728531	-0.665234469
P	1.705624326	0.689142421	2.805521206
P	1.787654569	0.424652123	-2.417438333
N	0.02831263	0.340951381	0.180027964
Cl	1.710848096	2.8905458	0.04100445
Cl	2.239981951	-1.743313223	0.362951722
Cl	4.576660484	0.8697143	0.215409926
C	1.025402845	2.308387166	3.626546874
C	2.093482522	3.420262718	3.524847904
C	0.635082597	2.101551678	5.106307401
C	-0.255421918	2.753286259	2.885741217
C	2.827448196	-0.291316739	4.046636571
C	3.874477617	0.679426796	4.635662306
C	1.998499484	-0.956309768	5.167075553
C	3.595674024	-1.404575043	3.306008227
C	0.175657428	-0.381083641	2.55742488
C	-0.616876736	-0.065697063	1.307631241
C	-2.005032098	-0.229682094	1.31273674
C	-2.756069255	0.009556333	0.1541635
C	-2.061233301	0.419662557	-0.992145016
C	-0.674561704	0.588026988	-0.960110896

C	-4.228684906	-0.101305541	0.172871004
C	-5.026820199	-0.727617704	1.187511019
C	-6.405715611	-0.550577737	0.865132632
C	-6.476096011	0.162065824	-0.375017569
C	-5.139871068	0.417756461	-0.807067132
C	-4.474136421	-3.295813629	-1.681471181
C	-5.210557602	-3.806911237	-0.574193684
C	-6.592897713	-3.500261605	-0.781260015
C	-6.703626736	-2.791167144	-2.022662786
C	-5.389193795	-2.665475563	-2.572963082
C	0.045201647	1.101682123	-2.188042533
C	2.66974511	1.647001017	-3.635224803
C	1.74187342	2.091979838	-4.786534634
C	3.938648294	0.957523815	-4.181471853
C	3.120360171	2.914013127	-2.879786617
C	1.5298097	-1.302861632	-3.25634537
C	1.139632981	-1.191344723	-4.746053518
C	2.825682908	-2.134118964	-3.12318363
C	0.373466191	-2.043979238	-2.547485359
H	3.004569954	3.179115045	4.079736594
H	2.367318447	3.638095111	2.488849735
H	1.680149189	4.339855189	3.961471377
H	-0.101154499	1.299582632	5.243456807
H	1.4906238	1.906195606	5.757562064
H	0.168595125	3.030760839	5.461794624
H	-0.565448108	3.722024419	3.3005298
H	-0.106970217	2.893950214	1.812958686
H	-1.088146126	2.057379706	3.048972775

H	4.462252845	1.175517753	3.853026272
H	3.442503688	1.443433563	5.287758748
H	4.57563431	0.094914311	5.2459516
H	2.691983126	-1.500953303	5.822425659
H	1.450452777	-0.249113739	5.792475089
H	1.290793235	-1.696358771	4.772135188
H	2.939454923	-2.187369375	2.914710324
H	4.207893395	-1.0191143	2.484629047
H	4.271810466	-1.878262961	4.030762641
H	0.557968005	-1.407813263	2.468353007
H	-0.482608917	-0.352180506	3.433240077
H	-2.487668828	-0.525093855	2.239607674
H	-2.58626143	0.624393568	-1.920751088
H	-4.654354626	-1.259241335	2.055906027
H	-7.24684132	-0.896862687	1.455631822
H	-7.379993158	0.46163903	-0.893992435
H	-4.869953834	0.942712634	-1.716203261
H	-3.396604571	-3.337532476	-1.800138313
H	-4.794857379	-4.324373281	0.283665052
H	-7.411219629	-3.763919634	-0.12017518
H	-7.62016792	-2.419716737	-2.467796811
H	-5.134291234	-2.162770278	-3.499675161
H	0.179275361	2.186389041	-2.070401708
H	-0.576661157	0.946371374	-3.076911369
H	2.296010185	2.807134165	-5.409890404
H	1.421632725	1.276700694	-5.438198542
H	0.851934953	2.618727158	-4.419189616
H	4.596004371	0.606955147	-3.375811407

H	3.723371844	0.11900384	-4.849443103
H	4.505076466	1.695720321	-4.764315611
H	2.281202045	3.509220593	-2.508006817
H	3.788118131	2.689650677	-2.042240091
H	3.676144361	3.542307613	-3.589230905
H	0.237533585	-0.586609263	-4.904074227
H	1.940299689	-0.79391685	-5.37463391
H	0.919061238	-2.203606091	-5.112877491
H	3.110073372	-2.291651955	-2.079154951
H	2.655219838	-3.120742699	-3.575896653
H	3.671448391	-1.67958358	-3.646721146
H	0.312184209	-3.055352794	-2.972980702
H	0.528952974	-2.151723631	-1.471706324
H	-0.594882347	-1.558177667	-2.727859216

Table S16. Cartesian coordinate of **IIb** in the triplet state. Units are presented in Å.

SCF energy = -5831.57514721 hartree

ZPE = 1.639646 hartree

Atom	X	Y	Z
Mo	2.348360183	1.016355093	0.332071382
Mo	-2.772426597	0.21901201	-0.113113572
Fe	9.961169447	-0.917324694	0.216241132
Fe	-10.7294084	-0.922181815	0.286692264
P	2.982258608	2.291730722	-1.882259406
P	2.870908286	-0.110695931	2.679070371
P	-3.86705668	2.550003691	0.30757011
P	-2.891659431	-2.32901107	-0.653256795
N	4.588381884	1.120436254	0.42891507
N	2.164869667	2.623166999	1.237737364
N	0.782937582	3.290569935	1.281978299
N	0.353076218	0.685509822	0.142810656
N	-0.801420682	0.503846997	0.062520127
N	-5.018124411	-0.155848006	-0.417158237
N	-2.619403998	0.707209013	-2.087203647
N	-2.530191054	0.992074907	-3.180556823
N	-2.882141396	-0.212415838	1.8662139
N	-2.922030767	-0.410739029	2.983593087
C	3.571723342	4.136452487	-1.707849817
C	2.399503046	5.064309323	-1.317622711
C	4.229891925	4.660200816	-3.00277865
C	4.618366285	4.25032243	-0.576807972
C	2.096053729	2.073336001	-3.592662193

C	1.110896572	3.2335265	-3.848795346
C	3.08469651	1.943603191	-4.770099538
C	1.257503549	0.780157127	-3.520731101
C	4.610424496	1.381441971	-2.043057658
C	5.321320442	1.196433414	-0.720055636
C	6.710080529	1.098398109	-0.706367251
C	7.415906019	0.93720223	0.497225401
C	6.641119545	0.911968909	1.670789457
C	5.253524595	0.987106561	1.611901926
C	8.874923755	0.823208651	0.531026782
C	9.775788177	0.974214678	-0.58720976
C	11.10802389	0.785292437	-0.120362063
C	11.05395021	0.499097057	1.278677684
C	9.688138822	0.514047464	1.682349592
C	8.675844024	-2.491041403	-0.240570727
C	9.633610548	-2.316541893	-1.28635209
C	10.93780129	-2.485120657	-0.724462267
C	10.78571619	-2.76245546	0.669665144
C	9.38709449	-2.764364077	0.969357573
C	4.435076419	0.918334085	2.881385036
C	1.85614029	0.353918863	4.264319895
C	2.738966906	0.523595493	5.519889672
C	0.756847546	-0.697766718	4.522376642
C	1.136490855	1.694793581	4.01681095
C	3.458541807	-1.935602457	2.833334184
C	3.88519518	-2.378058475	4.24740159
C	2.313017828	-2.83585438	2.323966432
C	4.687318186	-2.112509218	1.916402725

C	-3.149156088	4.217060434	-0.395298246
C	-2.160366214	3.850628648	-1.520027382
C	-2.363194622	4.976936619	0.691604572
C	-4.214051067	5.162039857	-0.994550462
C	-4.753851897	2.932391606	1.989100844
C	-3.702338419	3.022236264	3.117952314
C	-5.607634837	4.216301641	1.976965204
C	-5.703028229	1.756038439	2.317453955
C	-5.289389046	2.246310732	-0.878482194
C	-5.865814409	0.857628382	-0.749959268
C	-7.222241784	0.650308184	-0.982514585
C	-7.789149403	-0.630576859	-0.868010935
C	-6.900506255	-1.661657754	-0.520360453
C	-5.547836247	-1.407361134	-0.309584763
C	-9.209707324	-0.877831357	-1.125344684
C	-10.21603383	0.111807826	-1.42650888
C	-11.45890447	-0.554550573	-1.626948028
C	-11.24730729	-1.95557668	-1.443356642
C	-9.87367545	-2.159475426	-1.126743875
C	-9.935194162	-0.550391924	2.168472554
C	-10.91941302	0.435109907	1.845050365
C	-12.16101258	-0.238920406	1.622866586
C	-11.94337789	-1.639191976	1.808236019
C	-10.56708985	-1.832687989	2.144732923
C	-4.620230481	-2.536222909	0.056728166
C	-3.068442275	-2.905027372	-2.489730986
C	-1.777692064	-2.537911391	-3.255953685
C	-3.354765644	-4.411608803	-2.651912266

C	-4.255649744	-2.151930969	-3.132136575
C	-1.91373837	-3.677166269	0.340384618
C	-2.740860269	-4.950211241	0.626941245
C	-0.615286071	-4.052961825	-0.396727995
C	-1.520948153	-3.062904797	1.697033457
H	1.502427595	4.937292299	-1.929420323
H	2.147691951	4.918153084	-0.260764879
H	2.719301975	6.111640618	-1.414789635
H	5.05566346	4.021015237	-3.337160776
H	3.525812555	4.769603113	-3.832119201
H	4.654319014	5.654615101	-2.801966127
H	4.846073462	5.31646986	-0.427842652
H	4.241871182	3.847823112	0.368913906
H	5.561140393	3.753153027	-0.826960602
H	0.380351061	3.334282826	-3.035251089
H	1.596352397	4.201599312	-4.007257469
H	0.541603884	3.004824173	-4.76028514
H	2.50890732	1.79420768	-5.695057206
H	3.708267182	2.831806548	-4.913208746
H	3.735127469	1.069486046	-4.6540248
H	1.884228042	-0.111523484	-3.521831698
H	0.604606588	0.755370952	-2.641897113
H	0.618509196	0.728662073	-4.414197632
H	4.356997717	0.393631578	-2.455999151
H	5.286114131	1.863015751	-2.760126923
H	7.242205304	1.139894623	-1.652326617
H	7.120170488	0.819859393	2.641261305
H	9.49576552	1.192294401	-1.610680665

H	12.00317622	0.814135848	-0.730678568
H	11.90076014	0.269326641	1.914803732
H	9.329219692	0.304223089	2.682928707
H	7.600022346	-2.407897591	-0.345541796
H	9.411098988	-2.077051339	-2.319842752
H	11.87808722	-2.386351516	-1.254698573
H	11.59055353	-2.909774271	1.380876169
H	8.945975584	-2.925458404	1.946476431
H	4.074456754	1.926990952	3.127378744
H	5.059409635	0.576422016	3.71454608
H	2.094791748	0.779375753	6.373847404
H	3.286942458	-0.382966193	5.787174519
H	3.462497966	1.340477577	5.406279946
H	0.128479839	-0.859818199	3.637728814
H	1.156231966	-1.663511899	4.844368877
H	0.099660409	-0.335730782	5.326137191
H	1.836291471	2.499783578	3.75929901
H	0.396343241	1.586568613	3.215446722
H	0.602321954	1.983002505	4.933760728
H	4.684608813	-1.747994051	4.658658586
H	3.060561408	-2.407565876	4.966315023
H	4.286544542	-3.399766356	4.178744599
H	2.015627319	-2.566146992	1.306482884
H	2.656457075	-3.879517757	2.307959429
H	1.428918818	-2.788810286	2.966805415
H	4.903220386	-3.185564752	1.824732514
H	4.520004677	-1.721337295	0.91174677
H	5.577805763	-1.632730493	2.338886354

H	-1.67808808	4.769390934	-1.88692198
H	-1.382408114	3.158643389	-1.17548363
H	-2.65355941	3.381887824	-2.376992889
H	-1.803116336	5.799023136	0.221727858
H	-3.008091926	5.421994307	1.454971335
H	-1.642319686	4.326680855	1.205678862
H	-4.732377836	4.708048884	-1.846752137
H	-4.965652009	5.483327404	-0.268218563
H	-3.713881118	6.066748649	-1.370727817
H	-3.09016411	2.11703687	3.178859887
H	-3.040044368	3.887069716	3.012009838
H	-4.222293786	3.131614601	4.080700421
H	-6.155202878	4.285732071	2.928187831
H	-5.011266918	5.129581116	1.88451636
H	-6.35658612	4.211742781	1.174831707
H	-6.548626346	1.700822522	1.622830776
H	-5.195574577	0.78809607	2.321847727
H	-6.116288488	1.917638389	3.323607927
H	-6.086853545	2.993710638	-0.790307943
H	-4.851625102	2.361994169	-1.880537567
H	-7.838217851	1.499946412	-1.263338204
H	-7.258486047	-2.68164951	-0.413430367
H	-10.06576543	1.183616231	-1.478325889
H	-12.40704374	-0.076473728	-1.843824998
H	-12.00592778	-2.72752635	-1.499639469
H	-9.415264691	-3.118857123	-0.918613282
H	-8.884453965	-0.362616842	2.359777411
H	-10.75120433	1.502676856	1.76022382

H	-13.09584854	0.226668504	1.332407439
H	-12.68451298	-2.420337403	1.682603048
H	-10.08437487	-2.786090881	2.326857093
H	-5.074252657	-3.497482778	-0.212056924
H	-4.478058176	-2.543808118	1.147738122
H	-1.609362476	-1.457288591	-3.275796195
H	-0.876939285	-3.003160615	-2.84785062
H	-1.881868326	-2.873146347	-4.298433566
H	-2.51713887	-5.041427885	-2.339960734
H	-4.253398417	-4.729160508	-2.106435995
H	-3.535802584	-4.618703446	-3.716676015
H	-4.245888475	-2.350149875	-4.213452088
H	-5.221406817	-2.499414184	-2.746966754
H	-4.2017193	-1.068876466	-2.997510858
H	-2.105287371	-5.656145737	1.181155005
H	-3.614839674	-4.746846339	1.25787843
H	-3.080709802	-5.462165803	-0.276884501
H	-0.006756146	-4.685487694	0.266388976
H	-0.794879908	-4.630469884	-1.309101995
H	-0.011360702	-3.181680086	-0.66726756
H	-2.388851169	-2.80755687	2.313732041
H	-0.930319014	-3.802689775	2.256776552
H	-0.908290563	-2.164243486	1.569785306
S	2.909340109	-1.951905967	-1.908561561
O	1.557054173	-2.47162973	-2.16363895
O	3.671569337	-1.437475198	-3.068795296
O	3.000311286	-1.035182106	-0.697471735
C	3.911333128	-3.445061016	-1.401509236

F	5.172951098	-3.083508191	-1.096310448
F	3.372755697	-4.055449455	-0.332386294
F	3.947315809	-4.311318654	-2.41553022
H	0.770269408	4.077632615	0.622431938
H	0.040676622	2.622417651	1.020594801
H	0.612903681	3.649305248	2.229140088

References.

S1 Murphy, J. M.; Liao, X.; Hartwig, J. F. *J. Am. Chem. Soc.* **2007**, *129*, 15434.

S2 Datta, A.; Köllhofer, A.; Plenio, H. *Chem. Commun.* **2004**, 1508.

S3 Sato, M.; Maruyama, G.; Tanemura, A. *J. Organomet. Chem.* **2002**, *655*, 23.

S4 (a) Fleckenstein, C. A.; Plenio, H. *Adv. Synth. Catal.* **2006**, *348*, 1058. (b) Kadkin, O. N.; Kim, E. H.; Rha, Y. J.; Kim, S. Y.; Tae, J.; Choi, M.-G. *Chem. Eur. J.* **2009**, *15*, 10343.

S5 Kuriyama, S.; Arashiba, K.; Nakajima, K.; Tanala, H.; Kamaru, N.; Yoshizawa, K.; Nishibayashi, Y. *J. Am. Chem. Soc.* **2014**, *136*, 9719.

S6 Stoffelbach, F.; Saurenz, D.; Poli, R. *Eur. J. Inorg. Chem.* **2001**, 2699.

S7 Arashiba, K.; Miyake, Y.; Nishibayashi, Y. *Nat. Chem.* **2011**, *3*, 120.

S8 Chisholm, M. H.; D'Acchioli, J. S.; Hadad, C. M.; Patmore, N. J. *Inorg. Chem.* **2006**, *45*, 11035.

S9 Robbins, J. L.; Edelstein, N.; Spencer, B.; Smart, J. C. *J. Am. Chem. Soc.* **1982**, *104*, 1882.

S10 Kawatsura, M.; Hartwig, J. F. *Organometallics* **2001**, *20*, 1960.

S11 *CrystalStructure 4.0: Single Crystal Structure Analysis Software*; Rigaku Corp: Tokyo, Japan, and MSC: The Woodlands, TX, 2010.

S12 *SIR-97*: Altomare, A.; Burla, M. C.; Camalli, M.; Cascarano, G. L.; Giacovazzo, C.; Guagliardi, A.; Moliterni, A. G. G.; Polidori, G.; Spagna, R. *J. Appl. Crystallogr*. **1999**, *32*, 115.

S13 *SIR2002*: Burla, M. C., Camalli, M., Carrozzini, B., Cascarano, G. L., Giacovazzo, C., Polidori, G., Spagna., R. *J. Appl. Crystallogr*. **2003**, *36*, 1103.

S14 Beurskens, P. T., Beurskens, G., de Gelder, R., García-Granda, S., Gould, R. O., Israël, R. & Smits, J. M. M. *The DIRDIF-99 Program System*; Crystallography Laboratory, University of Nijmegen: Nijmegen, The Netherlands, 1999.

S15 Brunschwig, B. S.; Creutz, C.; Sutin, N. *Chem. Soc. Rev.* **2002**, *31*, 168.

S16 Weatherburn, M. W. *Anal. Chem*. **1967**, *39,* 971.

S17 Clarke, K.; Rothwell, K. *J. Chem. Soc.* **1960**, *1885*.

S18 Frisch, M. J.; Trucks, G. W.; Schlegel, H. B.; Scuseria, G. E.; Robb, M. A.; Cheeseman, J. R.; Scalmani, G.; Barone, V.; Mennucci, B.; Petersson, G. A.; Nakatsuji, H.; Caricato, M.; Li, X.; Hratchian, H. P.; Izmaylov, A. F.; Bloino, J.; Zheng, G.; Sonnenberg, J. L.; Hada, M.; Ehara, M.; Toyota, K.; Fukuda, R.; Hasegawa, J.; Ishida, M.; Nakajima, T.; Honda, Y.; Kitao, O.; Nakai, H.;

Vreven, T.; Montgomery, J. A., Jr.; Peralta, J. E.; Ogliaro, F.; Bearpark, M.; Heyd, J. J.; Brothers, E.; Kudin, K. N.; Staroverov, V. N.; Keith, T.; Kobayashi, R.; Normand, J.; Raghavachari, K.; Rendell, A.; Burant, J. C.; Iyengar, S. S.; Tomasi, J.; Cossi, M.; Rega, N.; Millam, N. J.; Klene, M.; Knox, J. E.; Cross, J. B.; Bakken, V.; Adamo, C.; Jaramillo, J.; Gomperts, R.; Stratmann, R. E.; Yazyev, O.; Austin, A. J.; Cammi, R.; Pomelli, C.; Ochterski, J. W.; Martin, R. L.; Morokuma, K.; Zakrzewski, V. G.; Voth, G. A.; Salvador, P.; Dannenberg, J. J.; Dapprich, S.; Daniels, A. D.; Farkas, O.; Foresman, J. B.; Ortiz, J. V.; Cioslowski, J.; Fox, D. J. *Gaussian 09*, Revision C.01; Gaussian, Inc.: Wallingford CT, 2010.

S19 (a) Yuki, M.; Tanaka, H.; Sasaki, K.; Miyake, Y.; Yoshizawa, K.; Nishibayashi, Y. *Nat. Commun.* **2012**, *3*, 1254. (b) Tanaka, H.; Arashiba, K.; Kuriyama, S.; Sasada, A.; Nakajima, K.; Yoshizawa, K.; Nishibayashi, Y. *Nat. Commun.* **2014**, *5*, 3737.

S20 (a) Becke, A. D. *Phys. Rev. A* **1988**, *38*, 3098-3100. (b) Becke, A. D. *J. Chem. Phys.* **1993**, *98*, 5648-5652. (c) Lee, C.; Yang, W.; Parr, R. G. *Phys. Rev. B* **1988**, *37*, 785-789. (d) Vosko, S. H.; Wilk, L.; Nusair, M. J. *Can. J. Phys.* **1980**, *58*, 1200.

S21 (a) Reiher, M.; Salomon, O.; Hess, B. A. *Theor. Chem. Acc.* **2001**, *107*, 48. (b) Reiher, M. *Inorg. Chem.* **2002**, *41*, 6928.

S22 Hay, P. J.; Wadt, W. R. *J. Chem. Phys.* **1985**, *82*, 299.

S23 (a) Ditchfield, R.; Hehre, W. J.; Pople, J. A. *J. Chem. Phys.* **1971**, *54*, 724. (b) Hehre, W. J.; Ditchfield, R.; Pople, J. A. *J. Chem. Phys.* **1972**, *56*, 2257. (c) Hariharan, P. C.; Pople, J. A. *Theor. Chem. Acc.* **1973**, *28*, 213. (d) Francl, M. M.; Pietro, W. J.; Hehre, W. J.; Binkley, J. S.; Gordon, M. S.; DeFrees, D. J.; Pople, J. A. *J. Chem. Phys.* **1982**, *77*, 3654.

S24 Dreuw, A.; Head-Gordon, M. *Chem. Rev.* **2005**, *105*, 4009.

S25 (a) Dolg, M.; Wedig, U.; Stoll, H.; Preuß, H. *J. Chem. Phys.* **1987**, *86*, 866. (b) Andrae, D.; Häußermann, U.; Dolg, M.; Stoll, H.; Preuß, H. *Theor. Chim. Acta* **1990**, *77*, 123-141.

S26 Miyazaki, T.; Tanaka, H.; Tanabe, Y.; Yuki, M.; Nakajima, K.; Yoshizawa, K.; Nishizayashi, Y. *Angew. Chem .Int. Ed.* **2014**, *53*, 11488.

S27 O'Boyle, N. M.; Tenderholt, A. L.; Langner, K. M. *J. Comput. Chem.* **2008**, *29*, 839.
